# Metal–Organic Framework-Based Photodetectors

**DOI:** 10.1007/s40820-024-01465-7

**Published:** 2024-07-25

**Authors:** Jin-Biao Zhang, Yi-Bo Tian, Zhi-Gang Gu, Jian Zhang

**Affiliations:** 1 State Key Laboratory of Structural Chemistry, Structure of Matter, Fujian Institute of Research, Chinese Academy of Sciences, Fuzhou, Fujian 350002 People’s Republic of China; 2 College of Chemistry and Materials Science, Fujian Nornal University, Fuzhou, 350007 Fujian People’s Republic of China; 3 Fujian Science & Technology Innovation Laboratory for Optoelectronic Information of China, Fuzhou, 350108 Fujian People’s Republic of China; 4 University of Chinese Academy of Science, Beijing, 100049 People’s Republic of China

**Keywords:** Metal–organic frameworks, Semiconductor, Photodetectors

## Abstract

The unique and interesting physical and chemical properties of metal–organic framework (MOF) materials have recently attracted extensive attention in a new generation of photoelectric applications. In this review, we summarized and discussed the research progress on MOF-based photodetectors. The methods of preparing MOF-based photodetectors and various types of MOF single crystals and thin film as well as MOF composites are introduced in details. Additionally, the photodetectors applications for X-ray, ultraviolet and infrared light, biological detectors, and circularly polarized light photodetectors are discussed. Furthermore, summaries and challenges are provided for this important research field.

## Highlights

- The methods of preparing metal–organic framework (MOF)-based photodetectors and various types of MOFs are introduced.
- The applications of MOF photodetectors in the detection of X-ray, ultraviolet, and infrared radiation, biosensing, and circularly polarized light detection are summarized.
- Challenges in developing practical MOF photodetector and concepts to solve those critical challenges are discussed.

## Graphical abstract

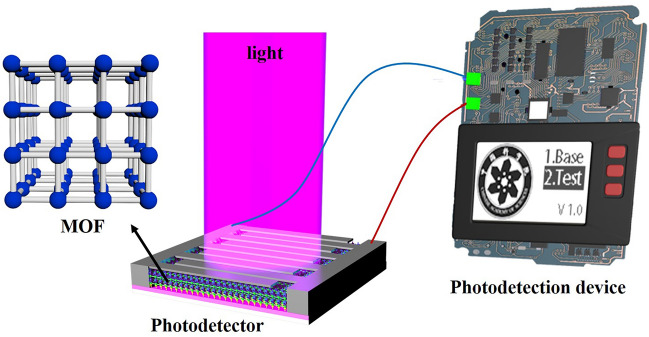

## Introduction

In this age of rapid information advancement, the ability of photodetectors to transform optical signals to electrical signals is crucial [1]. Photodetectors are extensively utilized in various fields of military and civilian applications, such as infrared guidance [2], night-vision device [3], space exploration [4], fire detection [5], ultraviolet communication [6, 7], medical imaging [8], video imaging [9] and security detection [10]. On the one hand, due to their high carrier mobility, low exciton binding energy and excellent stability, inorganic semiconductor photodiodes are currently the preferred choice for most photodetectors [11, 12]. Various inorganic materials such as GaAs, SiC, CuO and perovskite have been utilized for light detection across UV, visible and near-infrared systems [13–17]. On the other hand, organic semiconductors have emerged as promising candidates for the next generation of optical detection owing to their inherent advantages such as facile processing, mechanical flexibility, tunable absorption properties and low manufacturing costs [18–20]. Organic semiconductors refer to organic materials that exhibit semiconductor properties, primarily composed of organic molecular compounds or polymers [21]. In terms of structural characteristics, organic semiconductors exhibit weak van der Waals forces between molecules, while inorganic semiconductors are composed of covalent bonds between atoms. These differences in composition and structure result in significant variations in their mechanical and photoelectric properties [12]. By integrating the benefits of both inorganic and organic components, a novel approach for high-performance photodetectors is proposed to broaden their application scope.

Metal–organic framework (MOF), also known as porous coordination polymers, is a type of inorganic–organic hybrid material. They are formed by coordinating metal ions or clusters with organic bridge ligands to create porous crystalline structures. Due to its unique construction mode [22], diverse topological network structure [23, 24], and abundant post-synthetic modification sites [25, 26], MOF has found widespread use in separation [27–30], catalysis [31–34], sensing [35, 36], biological medicine [37, 38] and other fields. They possess a vast specific surface area, exceptional thermal stability, adjustable structure, and an abundance of active sites [39–41]. MOF is highly valued for their ability to adapt to the host through host–guest interactions, as well as their structural diversity and high porosity, which confer upon them a range of physical and chemical properties [42, 43]. The ligands in MOF absorb light and the photo-excited electrons can further inject into metal nodes to produce electron–hole pairs by ligands-to-clusters charge transfer, exhibiting extraordinary semiconductor-like behavior [44–46]. However, the vast majority of MOFs have low conductivity at room temperature, which hinders their application in optoelectronic devices where good charge transport performance is required.

MOF is generally considered as a wide bandgap insulating material. At present, various simulation techniques have been used to optimize its bandgap to the required value of semiconductors. Density functional theory (DFT) has been used to calculate the energy of HOMO–LUMO energy levels of most reported MOF periodic structures. Volkmer et al. [47] investigated the relationship between the crystal structure and bandgap of MFU-4-type MOF by combining experiments with calculations. The results found that the valence band (HOMO) energy could be increased by increasing the conjugation degree of ligands, thus reducing the bandgap. In addition, the energy level of the conduction band could be adjusted by selecting appropriate metals to regulate the bandgap. To further control the band gap, various functional groups (-OH, -NH_2_, -Cl, -CH_3_) can be used to modify the bridging ligands, which can provide electrons and lead to changes in the band gap. DFT calculations have shown that the bandgap for MIL-125 MOF decreased from 3.5 to 2.4 eV when the benzenedicarboxylate (BDC) linker was functionalized by methyl (BDC-CH_3_) and hydroxyl (BDC-OH) groups [48]. Therefore, the photoelectronic properties of MOFs, such as band structure, quantum efficiency, and carrier lifetime, are directly influenced by the composition and structure of MOFs [49]. Adjusting the chemical composition of organic linkers, edge functional groups, metal nodes, and polarity can significantly improve the performance of MOFs. Additionally, the morphology and arrangement of MOFs also affect their photoelectric properties.

**Fig. 1:**
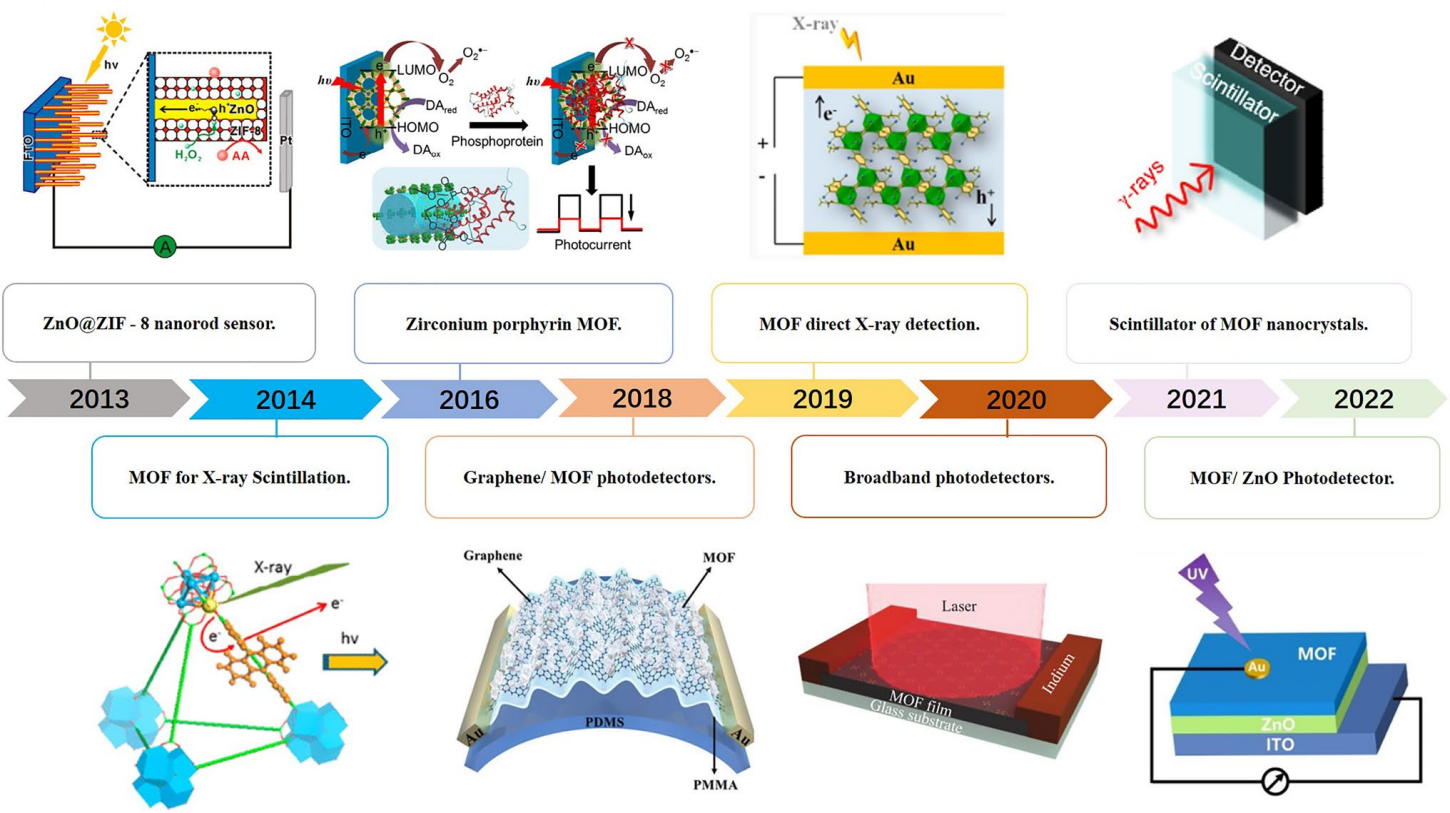
Schematic diagram of the discovery of MOF, material development and some major events in typical photoelectric applications

In this context, the research topic is still in its infancy, but notable advances and successful attempts to use MOF as active materials in optoelectronic devices (e.g., X-ray detectors, ultraviolet detectors, infrared detectors) have been reported in the past few years (Fig. 1). MOF is expected to become a better alternative to traditional semiconductor materials in the microelectronics industry due to its unique structural properties, tunability, advantages of organic and inorganic components, and versatility.

**Scheme 1:**
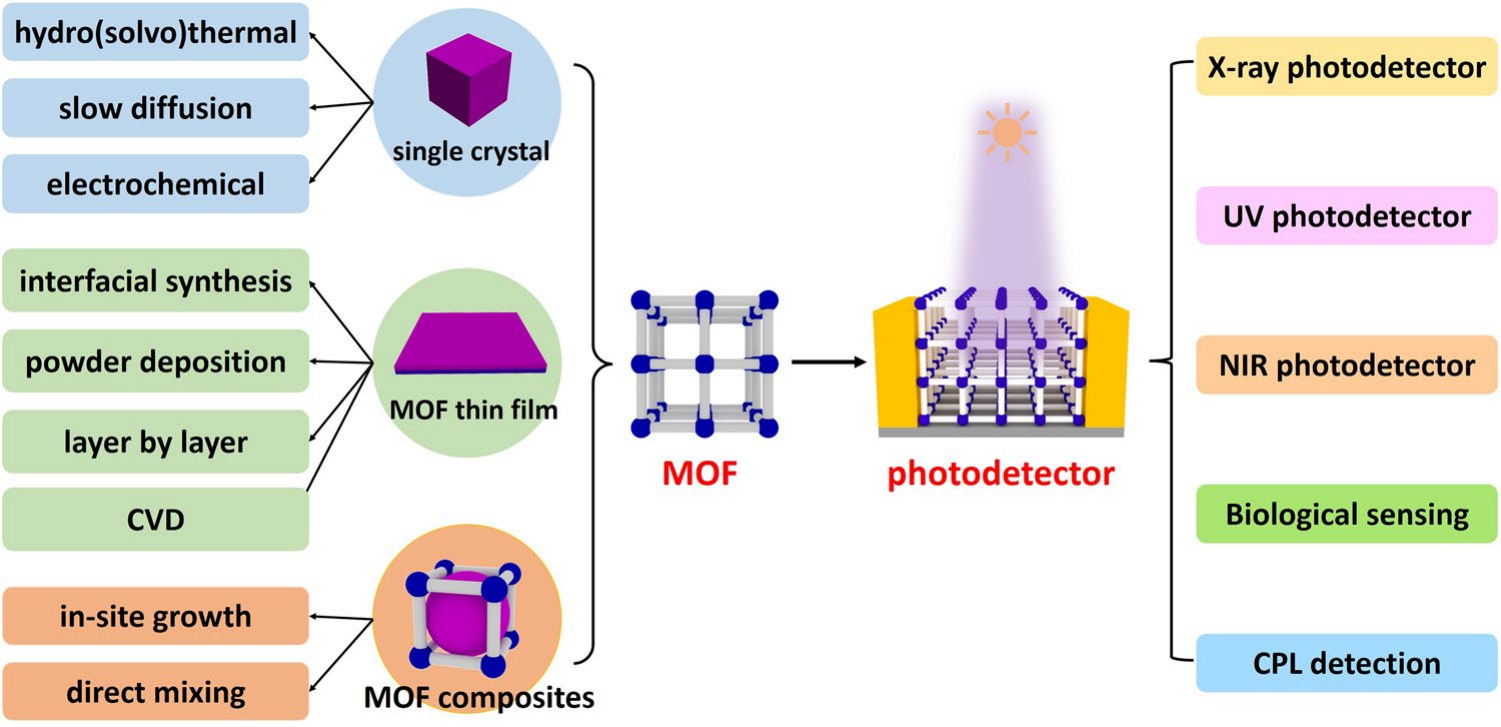
An overview of the synthetic strategies of MOF and the applications in photodetector

As an emerging and growth research field, it is necessary to summarize the reported MOF-based photodetectors and outlook their promising potential applications. In this review, the development of MOF-based photodetectors in recent years is discussed. We firstly introduce the classification and preparation methods of MOF-based photodetectors, and then discuss various photodetectors based on MOF single crystals, MOF thin films and MOF composites, with an emphasis on their light response properties (Scheme 1). Finally, we discussed the future possibilities of MOF-based photodetectors.

## Classification of MOF-Based Photodetectors

There are primarily three forms of MOF utilized to assemble MOF-based photodetectors: MOF single crystals, MOF thin films, and MOF composites.

### MOF Single Crystals

Single crystals are good candidates for optoelectronic devices due to their structural perfection without grain boundaries and favorable factors that influence the intrinsic conductivity of the materials [50]. MOF single crystals can be synthesized by many different methods, including solvothermal method, slow diffusion, hydrothermal, electrochemical, mechanochemical, microwave assisted heating, ultrasound and so on [51–53]. Currently, the MOF single crystals used to make photodetectors are mostly synthesized by hydro(solvo)thermal method.

#### Hydro(solvo) Thermal Method

**Fig. 2:**
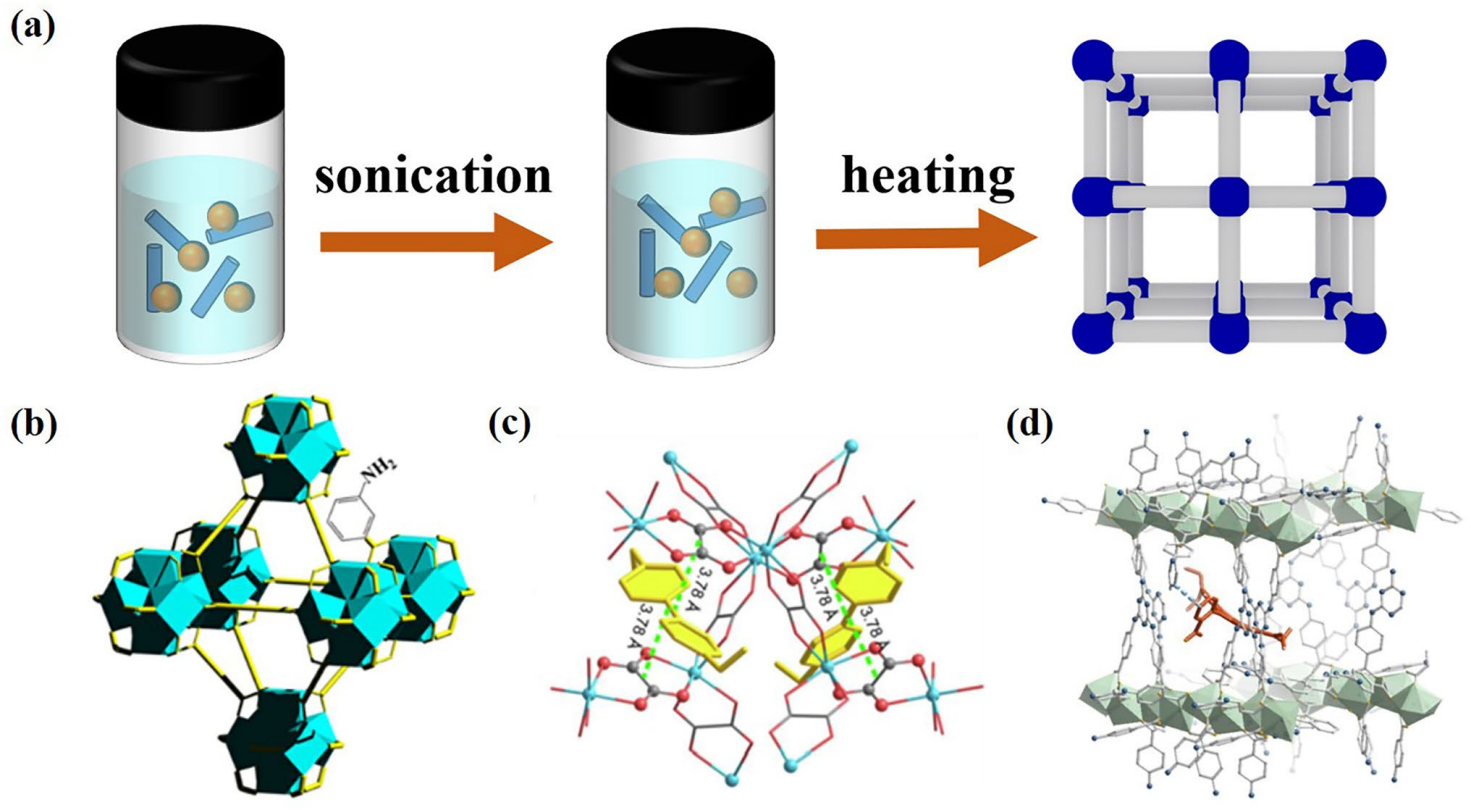
**a** Schematic diagram of solvothermal method. **b** Illustration of the crystal structures of UiO-66-NH_2_. Copyright 2021 Elsevier Ltd [54]. **c** Illustration of the crystal structures of (EV)[Zn_2_(ox)_3_] ∙3.5H_2_O. Copyright 2022 Elsevier Ltd [55]. **d** Illustration of the crystal structures of RhB^+^ @TbTATAB. Copyright 2022, American Chemical Society [56]

In this liquid phase synthesis process, a certain molar ratio of metals and ligands are added to the autoclave and allowed to react for a specific amount of time at a specific temperature (Fig. 2a) [57, 58]. Polytetrafluoroethylene lined autoclaves are usually used when reactants are at high boiling points. Depending on the kind of MOF crystal, a particular solvent will be employed. The commonly used solvents are DMF, DMSO, H_2_O and EtOH, and sometimes mixtures of these solvents are used. The MOF crystals synthesized by this method have good quality and are suitable for structural characterization [59]. Wang et al. [54] obtained nano-sized UiO-66-NH_2_ by hydrothermal method in the presence of benzoic acid (Fig. 2b). Then, they used UiO-66-NH_2_ as a self-calibration nanoprobe for the selective detection and biological imaging of hypochlorite. Under 400 nm excitation, UiO-66-NH_2_ has a luminescence at 432 nm and a new luminescence at 533 nm after adding ClO^−^ group. The emission intensity at 533 nm increases with the increase of ClO^−^ content. UiO-66-NH_2_ can effectively identify ClO^−^ by using the ratio of I_533_ nm/I_433_ nm as the detection signal. Guo et al. [55] successfully obtained a new MOF through a solvothermal reaction (Fig. 2c). This MOF represents the first rewritable radiochromic semiconductor material. When exposed to X-ray (such as K_α_ rays from Mo, Cu, Al anode), it shows photocurrent and clear color change. And Wang et al. [56] synthesized RhB^+^ @TbTATAB by solvothermal method (Fig. 2d).

### MOF Thin Film

Compared with powder or crystal MOF materials, MOF in thin films form has the advantages of continuous density and large specific surface area [60, 61], which can uniformly cover a larger range and they have greater practical value. High quality samples grown in thin film form are a good choice for integrating conductive MOF into optoelectronic devices [62–64]. Lu et al. [65] proposed that the basic problems of semiconductor MOF integration in active devices include thin film fabrication, contact formation and circuit design. Several techniques for growing MOF films on substrates have recently been developed. Many scientists are committed to the development of various synthesis methods of MOF thin films, and the following four methods are most often utilized in the fabrication of MOF thin films [66].

#### Interfacial Synthesis Method

**Fig. 3:**
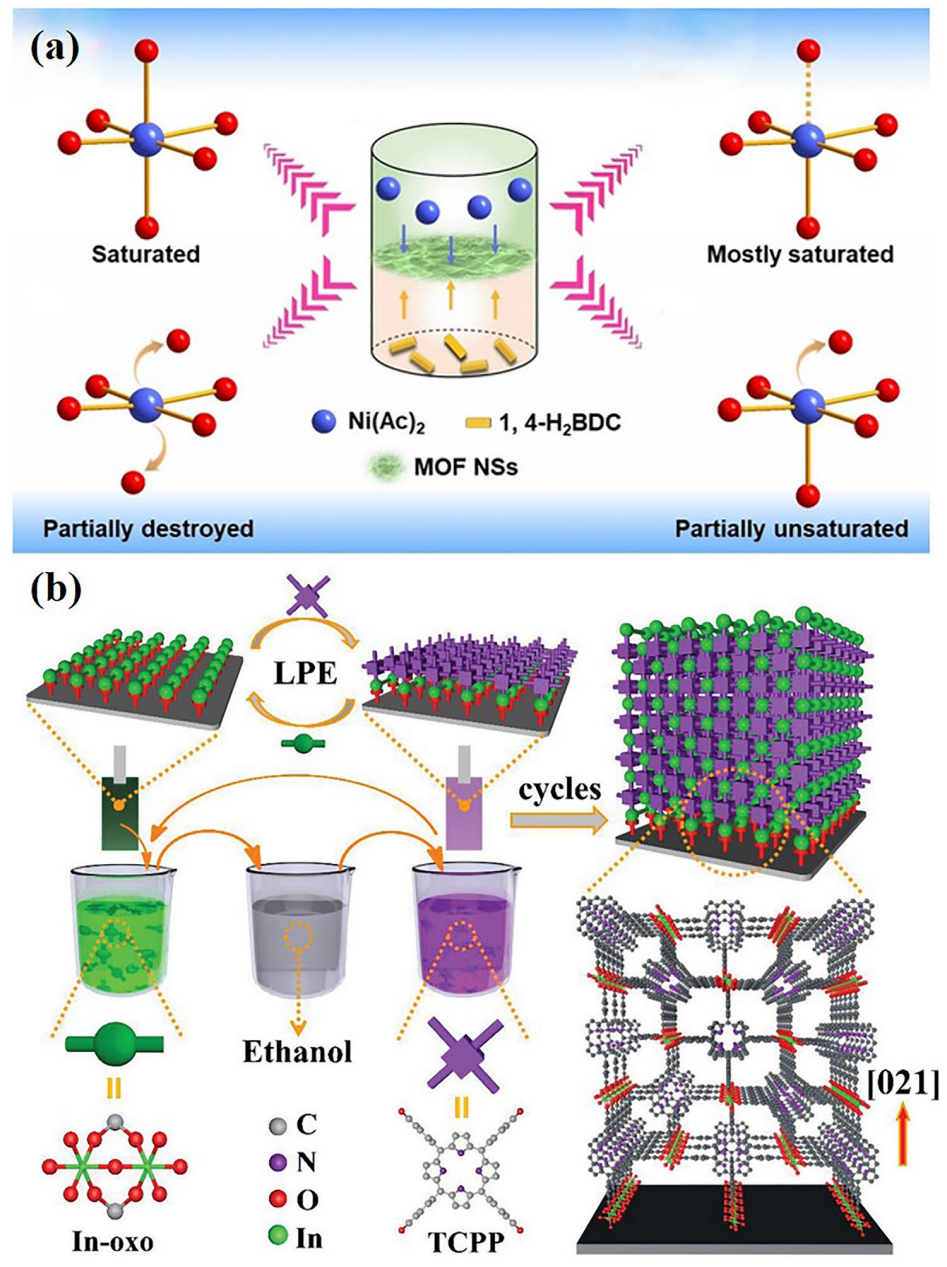
**a** Illustration of the preparation method for Ni MOF NSs through interfacial reaction. Copyright 2021, WILEY–VCH [68]. **b** Schematic illustration of MOF grown on functionalized substrate by using layer-by-layer dipping method. Copyright 2021, WILEY–VCH [69]

This technology uses the liquid/liquid or liquid/air interface to control the growth of MOF thin film. Because the reaction takes place at the solvent interface, the nucleation and growth of MOF can be well controlled. In particular, liquid/air interface synthesis can control the thickness of MOF thin films by utilizing the good dispersion of organic ligands on the liquid surface. This method has been widely used to prepare MOF thin films [67]. In 2021, Huang et al. have developed a mild liquid–liquid interfacial reaction method for the efficient synthesis of 2D Ni MOF nanosheets (NSs) with controlled molar ratios of metal precursors and organic linkers (*R*_M/L_), which can be directly used as effective and robust electrocatalysts for the two-electron oxygen reduction reaction (ORR) in alkaline solution. Figure 3a illustrates the method of preparing Ni MOF NSs by interface reaction [68].

#### Powder MOF-Based Deposition

This approach obtaining MOF thin films from the powder MOF. The facile deposition is achieved by direct drop-casting powder MOF onto appropriate substrates or prepatterned metal electrodes [66]. Kazemzad et al. [70] selected zeolite imidazolium framework (ZIF) with porous silicon (PS) loaded to form a porous MOF thin film. The ZIF/PS MOF thin film is used to manufacture semiconductor photodetector. It is found that the photocurrent is related to temperature and has significant photosensitivity to ultraviolet radiation at low temperatures.

#### Layer by Layer Method

The MOF thin film assembled by this method has the advantages of definite growth orientation, controllable film thickness and good uniformity. In order to prepare MOF thin films, the substrate should first be functionalized to provide coordination sites for MOF growth. Then, the functional substrate was immersed in the coordination of metal salt solution and metal nodes, and then the substrate was washed with solvent, then the organic ligand solution was immersed in the ligand for further coordination assembly, and the sample was washed again with solvent, so that the MOF thin film could be prepared [71–78]. In 2021, Xu et al. [79] demonstrated film chemically resistant sensors based on a conductive MOF using a spray LPE method. Highly oriented, ultra-thin, and low-roughness EC-MOF thin film was prepared and demonstrated as an effective interlayer material to modulate the height of the Schottky barrier. In 2022, Yan et al. [80] prepared a flexible photodetector based on Cu_3_(HHTT)_2_ thin film, which showed a reliable light response in the wavelength range from ultraviolet to mid infrared at room temperature. This is much wider than the previously reported solution treated broadband photodetectors. Recently, our group prepared In-TCPP films on SiO_2_/Si substrates by liquid phase epitaxy (LPE) layer by layer (LBL) method (see Fig. 3b), and further assembled photodetectors. The photoelectric detector has better light detection performance with a short rise/fall time (0.07/0.04 s) and a large detectivity (D*) of 7.28 × 10^14^ Jones [69].

#### Chemical Vapor Deposition (CVD)

Chemical vapor deposition (CVD) technology utilizes a top-down approach to deposit vaporized solid materials on various substrates to grow thin films. The vapor-phase growth for MOF thin films is achieved by converting the pre-deposited metal oxide layer into the corresponding MOF structure during the CVD deposition process of ligands. The CVD method can provide high-quality, directionally grown thin films that are not damaged by solvents, offering a uniform thickness of low-porosity coatings even on surfaces of various shapes [81]. This presents new opportunities for integrating semiconductor MOF into practical devices. For example, Park et al. [82] synthesized large-area, highly oriented two-dimensional conductive MOF films (Cu_3_(C_6_O_6_)_2_) using the CVD method. Subsequently, they employed electron beam lithography to create thin-film microdevices with a conductivity of up to 92.95 S cm^−1^.

### MOF Composites

A versatile MOF possesses tailorable outstanding optical property through an inherent multiple charge-transfer mechanism between metal and ligand. Yet, its commercialization remains unsuccessful because of its large porosity, poor conductivity, and deficient crystallinity [83]. It is possible to combine MOF with other materials to produce composites with better performance. The general synthesis method of MOF composites is to grow MOF thin films on the composite materials such as ZnO and graphene [83–87].

**Fig. 4:**
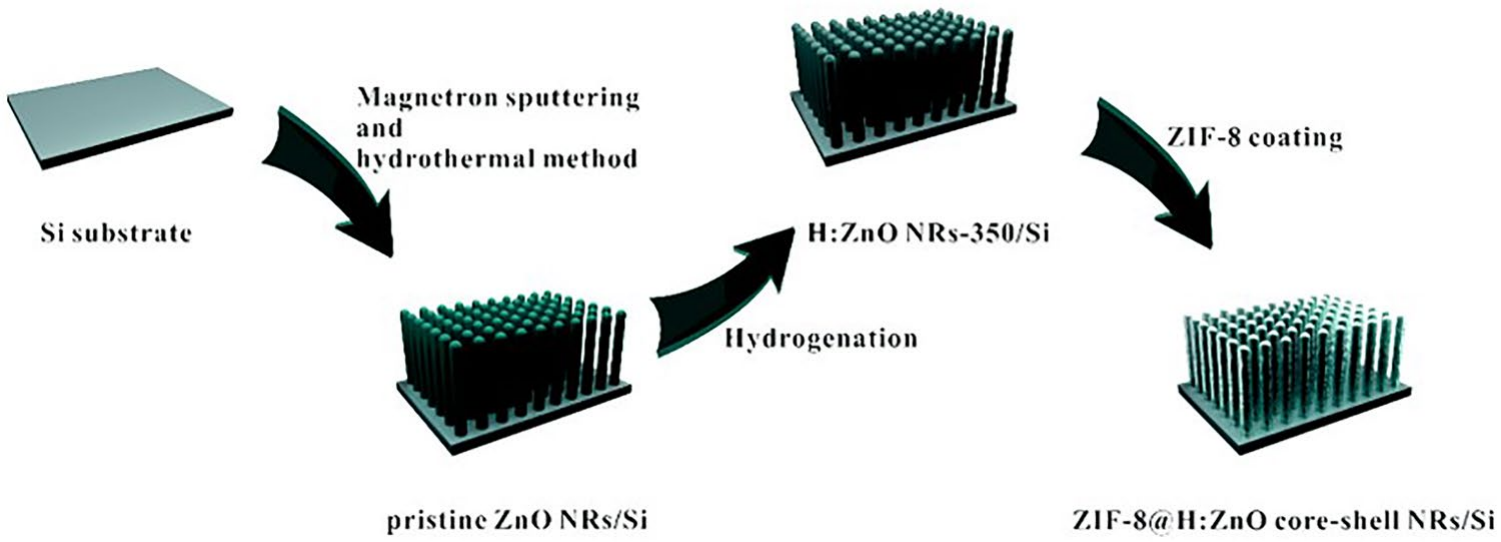
Illustration of the preparation method for ZIF-8@H:ZnO NRs/p-Si heterojunction. Copyright 2020, The Royal Society of Chemistry [86]

Recently, in order to resolve the slow sensing and the unexpected response to the visible light caused by the surface defects of ZnO, Xue et al. [86] assembled a ZIF-8@ZnO core–shell nanorod array/Si heterojunction self-powered photodetector. Figure 4 shows synthesis method of MOF and ZnO composites. Then, a photodetector is made by combining the Schottky junction on the top electrode of ZIF-8/Si. By hydrogenation and ZIF-8 combined treatment, its response rate is nearly 5 orders of magnitude higher than that of the original ZnO nanorods array/Si heterojunction photodetector. The photoresponse characteristics of the photodetector are significantly enhanced. The photodetector has a high detection rate of ~ 2.14 × 10^16^ Jones, a high response rate of ~ 7.07 × 10^4^ mA W^−1^. It can be comparable to self-powered photodetectors such as two-dimensional (2D) materials and zero-dimensional (0D) materials. This method of obtaining high-performance self-powered photodetectors through post-processing strategies has important application potential.

### Brief Summary of MOF Synthesis

Although these methods have been applied to manufacture photodetectors, they have their shortcomings in the practical applications. For instance, the hydro(solvo)thermal method, though simple in operation, is uncontrollable in terms of the size and quantity of the crystals obtained. While the interface synthesis method can produce high-quality films, its complex operation makes it difficult for industrial application. The powder deposition method is simple and suitable for most MOFs, but the films obtained are of poor quality with uneven surfaces. The layer by layer method can produce MOF films with oriented growth, controllable film thickness and good uniformity, but only a few MOFs can be grown by this method. The CVD technique can be used to deposit vaporized solid materials on various substrates to grow films without being affected by solvents, but it may inhibit growth by itself, making it difficult to grow relatively thick MOF films. We expect that with the development of research, more systematic and practical methods for preparing MOF-based photodetectors will be developed and extended to functional materials.

## Sensing and Devices Applications

MOF materials have recently generated several important optoelectronic device applications due to their excellent physical and chemical property, such as X-ray photodetector, UV/vis photodetector, NIR photodetector, and electrochemical detection in bioanalysis. MOF-based photodetector has achieved significant development over the past few years and many researchers are paying increasing attention to this prominent subgroup.

### X-Ray Photodetector

X-ray testing plays an important role in many fields, such as national, defense, medical diagnosis, nondestructive testing, and the nuclear industry [88–90]. The current flat panel detectors have a phenomenon called vignetting in non-planar scenes, so multiple X-ray exposures are used to ensure the imaging quality, which may cause radiation to the human body [91]. Therefore, it is expected to develop a flexible detector with minimum exposure time and frequency.

**Fig. 5:**
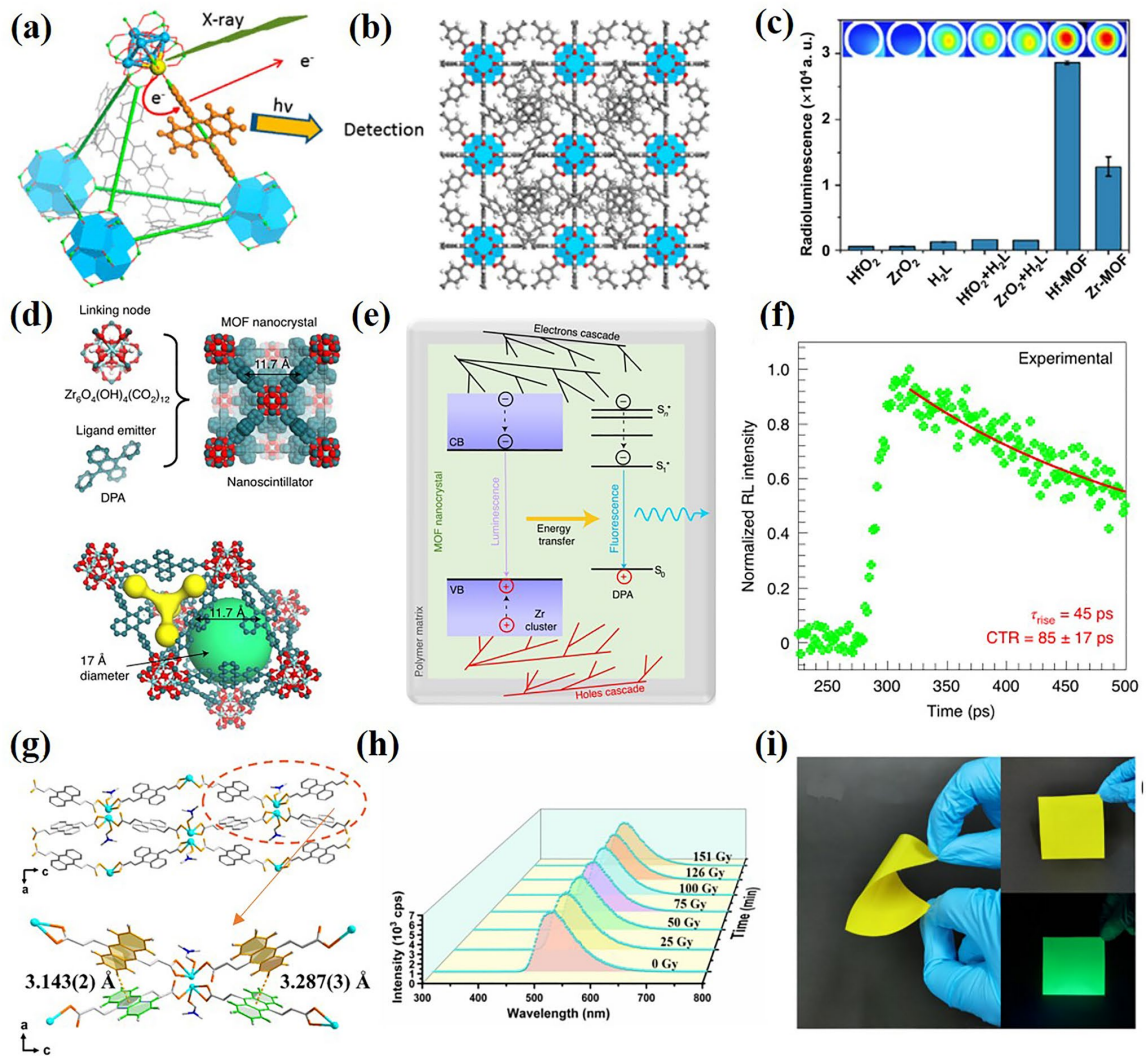
**a** Scintillation mechanism of the Hf-MOF and Zr-MOF. **b** Corresponding structural models viewed from the [99] direction. Blue polyhedra: Hf^4+^ or Zr^4+^ with eight coordinating oxygen atoms; red ball: oxygen; gray ball: carbon; white ball: hydrogen. **c** Radioluminescence signals of Hf-MOF, Zr-MOF and raw materials. Copyright 2014, American Chemical Society [94]. **d** Crystal structure of the Zr-based MOF. **e** Overview of photophysics involved in scintillation process. **f** Rise under pulsed X-ray excitation. Copyright 2021, Spring Nature Ltd [95]. **g** Crystal structure of [Pb(adda)(DMF)]_n_. **h** RL spectra of [Pb(adda)(DMF)]_n_ after different X-ray dose irradiation.** i** Photographs of a flexible Pb-MOF film. Copyright 2022 Elsevier Ltd [96]

X-ray detectors are mainly divided into indirect-type and direct-type X-ray detectors [92]. In the X-ray detector based on indirect conversion, the X-ray is first converted into light through a scintillating phosphor and then the light emitted from the scintillator is detected by the photodiode array [93]. Recently, MOF has been found to have potential applications in X-ray detection due to their high sensitivity, fast response time, high absorption coefficient, and radiation stability [92]. In 2014, Lin et al. [94] reported the X-ray excitation luminescence of MOF for the first time. Using high-Z metal clusters as connection nodes and anthracene-based emitters as bridging ligands, MOF was synthesized (Fig. 5b). Since Hf has a larger X-ray scattering cross section than Zr, Hf-MOF emitted a stronger signal than Zr-MOF (Fig. 5c). In addition, due to the synergistic effect of heavy metal clusters as X-ray antennae and bridging ligands as light emitters, MOF shows excellent X-ray to light conversion ability compared with the component itself (Fig. 5a). By use the same organic linker and a Zr-based metal cluster (Zr_6_O_4_(OH)_4_(CO_2_)_12_), Monguzzi et al. [95] reported a composite scintillator based on MOF nanocrystals embedded in polymer matrix (Fig. 5d). Because of the interaction between ionizing radiation and complex components, the singlet molecular exciton produced on the ligand is sensitized, and fluorescence is generated through radiation recombination with good radioluminescence stability (Fig. 5e). The nanocomposites show an ultrafast scintillation rise time of ~ 50 ps and mainly unaffected by light scattering (Fig. 5f). Currently, Guo et al. [96] synthesized X-ray responsive Scintillating MOF (SMOF) using another anthracene nuclear derivative (2E, 2E)-3,3'-(anthracene-9,10-diyl) diacrylic acid (H_2_adda) as an organic linker with good radioluminescence stability (Fig. 5g, h). Furthermore, the flexible scintillator film (Fig. 5i) based on MOF has been applied to X-ray imaging for the first time, achieving a high spatial resolution of 5.5 lp mm^−1^. This work provides an effective visualization tool for X-ray imaging and demonstrates the potential applicability of flexible MOF-based scintillator in X-ray imaging.

Except for the anthracene core derivatives, naphthalene core derivatives were also used as the organic luminescence linkers [92]. In 2018, Guo et al. [97] reported that the crystal type SMOF was constructed with Pb (II) ion as the functional motifs to absorb X-ray and naphthalene dicarboxylate (ndc^2−^) as the luminescent emitter. The Pb (II)—MOF scintillators have become candidate materials for X-ray detection due to the synergistic effect of Pb (II) center as effective X- ray absorbers and organic ligands as luminescent elements. Later, Guo group also published a series of Pb-based and Ba-based MOF scintillators using naphthalene core derivatives as organic linkers, which greatly enriched the materials and structures of MOF scintillators [98, 99].

**Fig. 6:**
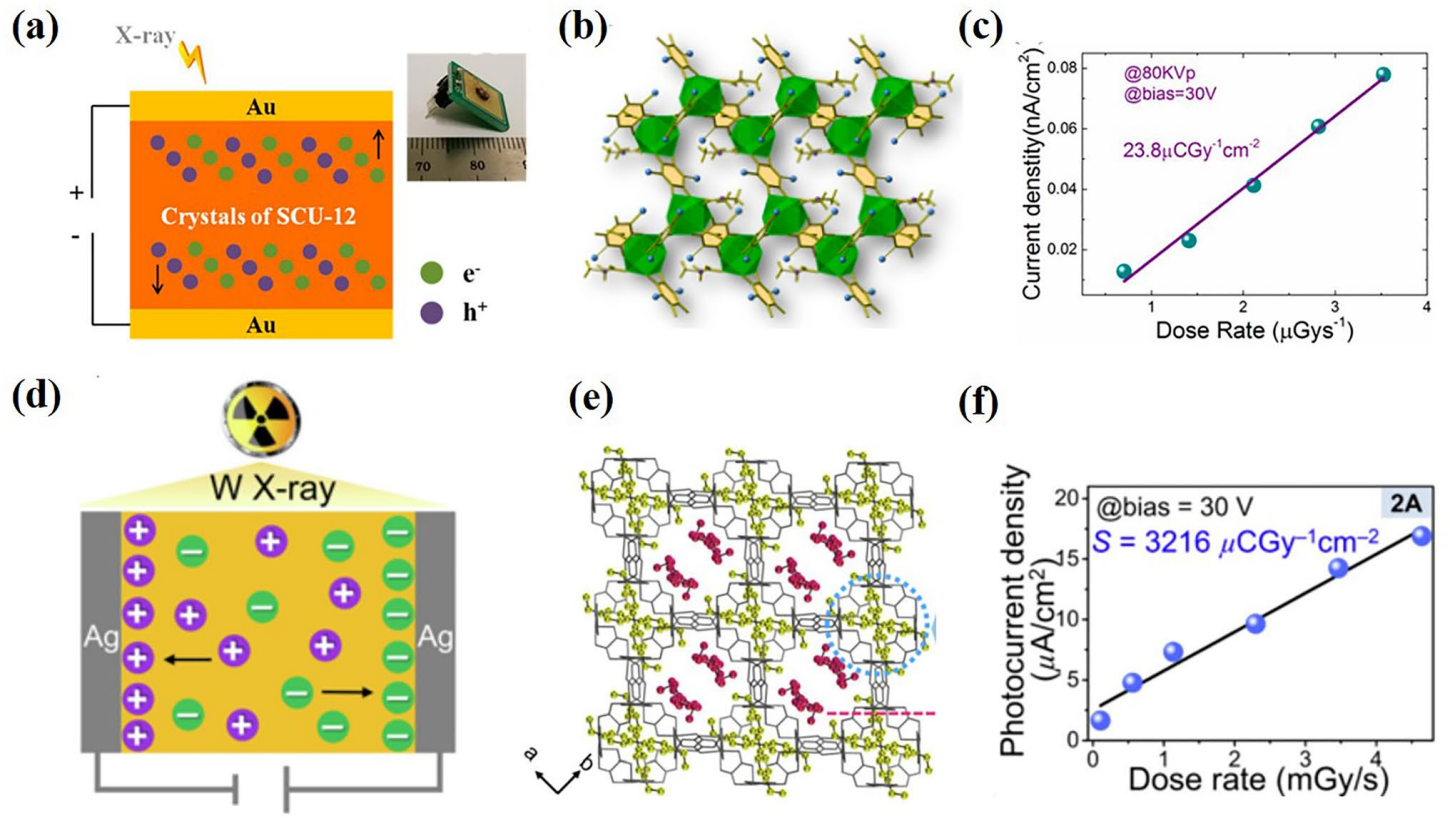
**a** Schematic illustration of SCU-12-based detector. Inset: the picture of detection device. **b** Illustration of the crystal structure of SCU-12. Color code: Tb (green), Cl (blue), benzene ring (yellow). **c** X-ray-generated photocurrent versus dose rate. Copyright 2019, American Chemical Society [100]. **d** Schematic diagram of experimental device of (EV)[Zn_2_(ox)_3_] ∙3.5H_2_O. **e** Schematic diagram of crystal structure of (EV)[Zn_2_(ox)_3_] ∙3.5H_2_O. **f** Photocurrent density versus dose rate measured at a bias voltage of 30 V for 2A. Copyright 2022, Elsevier Ltd [55]

Compared with indirect detection materials based on scintillators, semiconductors can directly convert X-ray photons into carriers, which has the advantages of improved energy and spatial resolution [93]. Wang et al. [100] investigated the direct radiation detection of semiconductor MOF. They show that a lanthanide-based semiconductive MOF (Fig. 6b) can effectively convert X-ray photons to electrical current signals under continuous hard X-ray radiation (Fig. 6a). The X-ray sensitivity of polycrystalline detection device based on semiconductor MOF under 80 kV_p_ X-ray irradiation is 23.8 μC Gy_air_^−1^ cm^–2^ (Fig. 6c), which can compete with amorphous selenium detectors on the market. The detector has the characteristics of a low detection limit of dose rate and good radiation stability under working conditions. Guo et al. [55] reported for the first time the use of rewritable radiochromic semiconductor MOF materials to make X-ray detectors (Fig. 6d, e). The sensitivity of semiconductor single crystal detector reaches 3216 µC Gy^–1^ cm^−2^ (Fig. 6f), which is higher than all reported MOF-based detectors and commercial detectors α- Se detector.

**Fig. 7:**
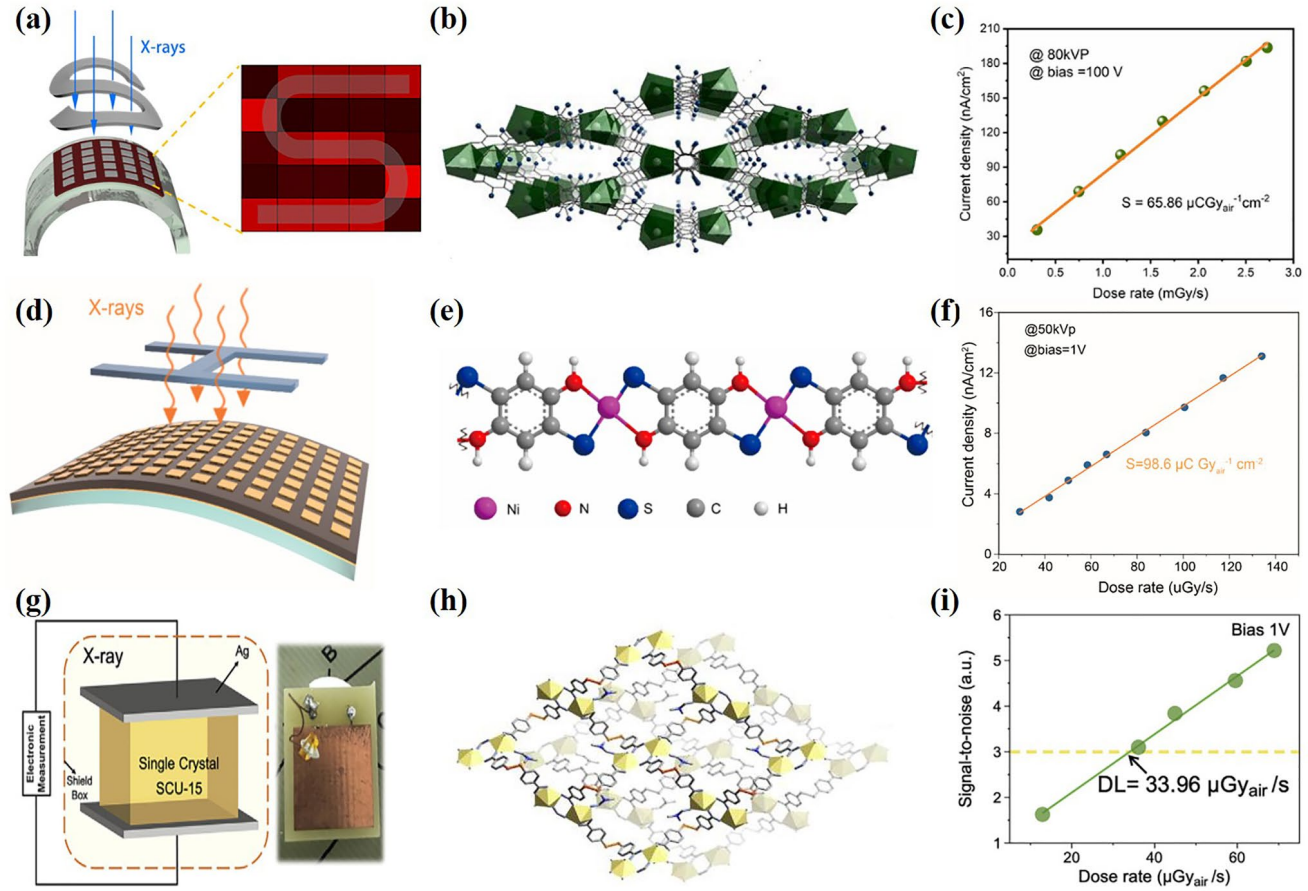
**a** Schematic illustration of a proof-of-concept flexible X-ray imager to image a curving “S”-shaped lead plate. **b** Illustration of the crystal structure of SCU-13. Color code: Pb (green), Cl (blue), benzene ring (black). **c** X-ray generated photocurrent versus X-ray dose rate of the membrane-based detector. Copyright 2020, WILEY–VCH [101]. **d** Schematic structure of X-ray prototype imaging device based on the Ni-DABDT MOF. **e** Crystal structures of the Ni-DABDT MOF. **f** Plot of dose rate dependence of X-ray generated photocurrent. Copyright 2021, American Chemical Society [102]. **g** Schematic of the device under X-ray irradiation. **h** Crystal structures of SCU-15. **i** Signal-to-noise ratio as a function of the X-ray dose rate. Copyright 2022, Elsevier Ltd [90]

Interestingly, some researchers have used semiconductor MOF for flexible electronic devices. In 2020, Wang et al. [101] manufactured a new generation of flexible X-ray detectors (Fig. 7a) by dispersing the semiconductive MOF [(CH_3_)_2_NH_2_]_2_PbL_2_(L = C_6_Cl_2_O_4_^2−^)] (Fig. 7b) based on heavy metals into polyvinylidene fluoride thermoplastic. They choose the element Pb with high atomic number as the metal node to ensure high X-ray attenuation efficiency. The redox activity of the ligand makes it possible to transport electrons in the whole framework. Polyvinylidene fluoride (PVDF) was chosen as the continuous phase to disperse the MOF crystals to make flexible photodetectors. A superior X-ray detection sensitivity of 65.86 µC Gy_air_^−1^ cm^−2^ is achieved (Fig. 7c). In addition, the device has excellent flexibility. After 500 bending cycles, its photocurrent degradation is very small. In 2021, Ren et al. [102] reported a flexible X-ray detector (Fig. 7d) using Ni-DABDT (DABDT = 2,5-diamino-1,4-benzenedithiol dihydrochloride) MOF (Fig. 7e) as the absorption layer. By directly converting X-ray photons into carriers, the detector has extremely high performance with a high detection sensitivity of 98.6 μC G_yair_^−1^ cm^−2^ (Fig. 7f), with a low detection limit of 7.2 μG_yair_ s^−1^ for the radiation robustness. The detector opens up a new opportunity to realize a high-sensitivity large-area X-ray imager with low-Z (atomic number) MOF.

Exciton behavior plays an important role in the photoelectric properties of semiconductor materials, but it remains to be explored in the MOF [56, 103]. Wang et al. [56] indicated that the exciton behavior in semiconductor MOF can be regulated by framework- guest interaction. The binding of electron defect molecules in the pores of terbium-based semiconductor MOF leads to effective energy transfer from MOF skeleton to molecular receptor. This energy transfer from MOF skeleton to molecular receptor. This interaction promotes the unique exciton type conversion, resulting in improved conductivity and photoelectric properties. The photocurrent on/off ratio changed from 1.2 to 79.5 after exciton conversion. The device reaches a sensitivity of 51.9 μC Gy_air_^−1^ cm^−2^ and an extremely low detection limit of 4.42 μGy_air_ s^−1^.

An important problem in the design of semiconductor MOF is to grow high-quality single crystals large enough for device fabricate [90]. In 2022, Wang et al. [90] adjusted the crystal morphology by controlling the reaction to obtain SCU-15 crystals of different sizes and used the large crystal material of SCU-15 to fabricate a single crystal X-ray detector (Fig. 7g, h), and further constructed a planar 2 × 3 pixel detector to show its potential application in X-ray imaging. Good crystal quality makes the detection performance of SCU-15 single crystal device superior to that of particle powder device. The detection sensitivity of the detector is 33.96 μG_yai_ s^−1^ (Fig. 7i) and has good stability. Table 1 summarizes the device performance of X-ray photodetectors based on MOFs.

**Table 1:** Device performance of X-ray photodetector based on MOFs

MOF	Metal; linker	Type	Preparation methodology	Application	Sensitivity (μC Gy_air_^−1^ cm^–2^)	References
Zr/Hf- MOF	M_6_(μ_3_-O)_4_(μ_3_-OH)_4_ (carboxylate)_12_;9,10-anthacenyl benzoic acid	Crystal	Solvothermal	scintillator	–	[94]
Zr-MOF	Zr_6_O_4_(OH)_4_(CO_2_)_12_;	Crystal	Solvothermal	scintillator	–	[95]
Pb-MOF	PbCl_2;_ H_2_adda	Film	knife coating	scintillator	–	[96]
SMOFs	PbCl_2;_ 1,4-H_2_ndc	Crystal	Solvothermal	scintillator	–	[97]
Ba-MOF	BaCl_2;_ naphthalene disulfonates	Crystal	Solvothermal	scintillator	–	[98]
Pb-sMOFS	PbCl_2_/PbBr_2_; 2,6-H_2_ndc	Crystal	Solvothermal	scintillator	–	[99]
SCU-12	TbCl_3_; chloranilic acid	Crystal	Solvothermal	semiconductors	23.8	[100]
Zn-MOF	Zn(NO_3_)_2_	Crystal	Solvothermal	semiconductors	3216	[58]
SCU-13	PbCl_2_; chloranilic acid	Crystal	Solvothermal	semiconductors	65.86	[101]
Ni-DABDT MOFs	NiCl_2_; DABDT	Crystal	Solvothermal	semiconductors	98.6	[102]
TbTATAB	Tb(NO_3_)_3_; H_3_TATAB	Crystal	Solvothermal	semiconductors	51.9	[59]
SCU-15	UO_2_(NO_3_)_2_; 4-MBA	Crystal	Solvothermal	semiconductors	3.51	[90]

### UV/Vis Photodetector

The detection of ultraviolet (UV) radiation is very important in scientific, commercial, environmental and medical fields [104–106]. For example, excessive exposure to ultraviolet radiation will have an impact on human health [107]. Additionally, UV light may cause a decline in animal populations [108]. Generally, humans cannot observe ultraviolet light with the naked eye, so it is worthwhile to develop an efficient UV photodetector. MOF has suitable ultraviolet absorption, tunable properties, and good compatibility, making them attractive for a wide range of ultraviolet applications.

**Fig. 8:**
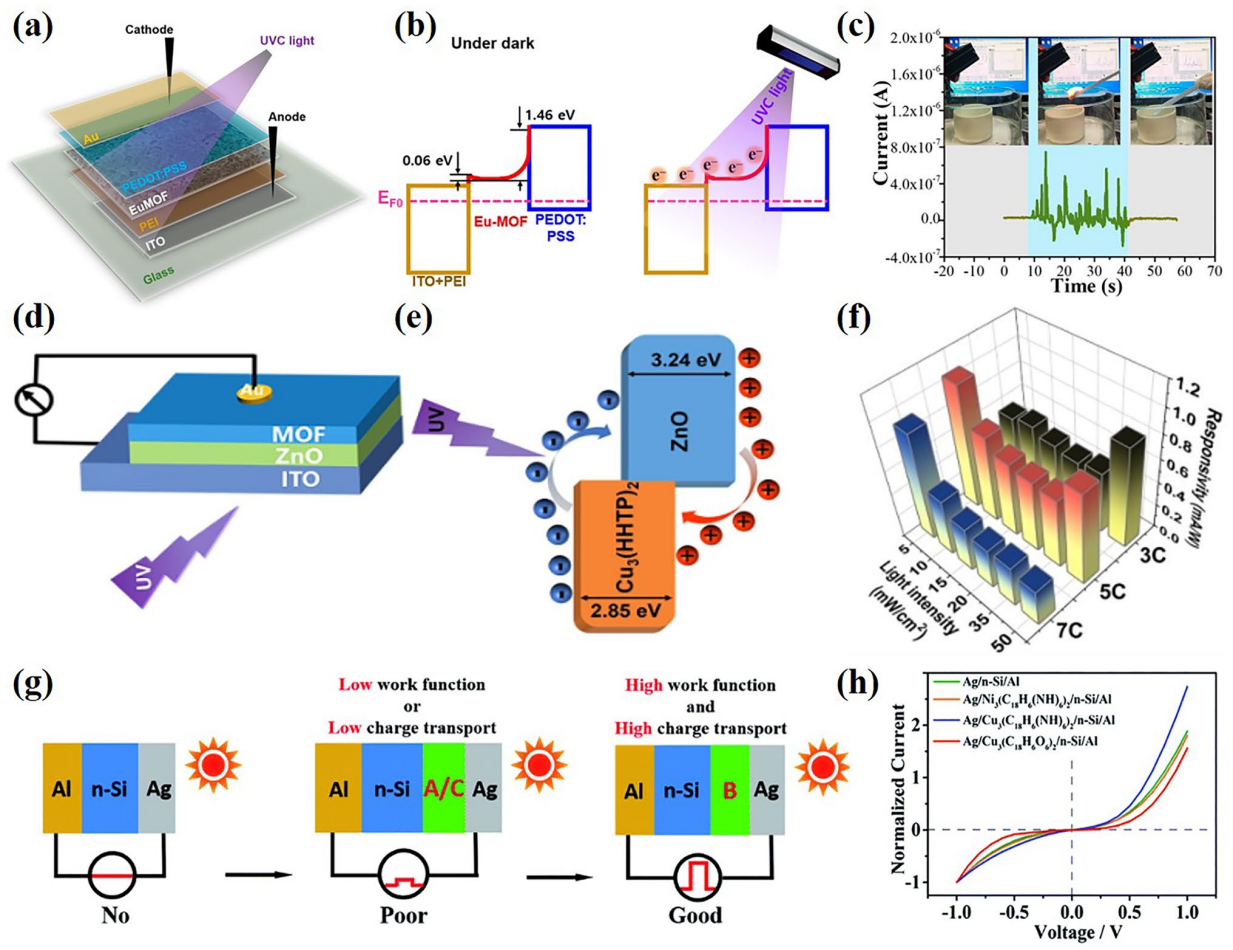
**a** Schematic diagram of ultraviolet photodetector. **b** Transport charge carriers in darkness and UVC illumination. **c** Current signal recorded during indoor methanol ignition. Copyright 2022, American Chemical Society [110]. **d** Schematic diagram of the device structure under the incident light from the bottom of the device.** e** Energy band diagram of the Cu_3_(HHTP)_2_/ZnO heterojunction. **f** Responsiveness of devices under different lighting power densities at a zero bias. Copyright 2018, WILEY–VCH [85]. **g** Device construction and photoelectric performance illustration of Ag/EC-MOF/n-Si Schottky diodes. **h** Device current–voltage diagram. Copyright 2020, The Royal Society of Chemistry [79]

Lanthanide metal–organic frameworks (Ln-MOF) have significant structural diversity and show interesting physical and chemical properties in the field of photodetection. Since Wang et al. [109] developed the first lanthanum-based photodetector to monitor ultraviolet radiation, Bark et al. [110] reported the application of Eu-MOF as core material in selective UVC detector (Fig. 8a). Eu-MOF is used as broadband gap light absorber in UVC PD (Fig. 8b). The device has good response to 254 nm ultraviolet radiation, self-powered operation, high switching ratio of 107.33, fast response time of 98/122 ms (rise/fall time), and strong inhibition ability to UVA and visible light. In addition, the unpacked UVC PD still maintains almost constant light response after being stored in the air for one month. In practical application, Eu-MOF photodetector can effectively measure the ultraviolet radiation of methanol fire under zero bias voltage (Fig. 8c). By doping europium into MOF, Junior et al. [42] reported the solvothermal synthesis and structural characterization of new luminescent MOF [Zn(BDC)(dpNDI):x%Eu^3+^ (x = 1, 2 and 5)] obtained from europium-doping (1%, 2%, and 5%). The MOF demonstrated reversible detection capability for radiation dose monitoring after excitation at 295 nm with a decrease in the intensity of the emission signal of the luminescence spectrum. Recently, Zeng et al. [85] demonstrated a Cu_3_(HHTP)_2_/ZnO type-II heterojunction UV photodetector fabricated by layer-by-layer (LBL) deposition. The photodetector was fabricated with a vertical structure (Fig. 8d) and Fig. 8e shows the movement of carriers in the device. The device achieves a responsivity of 78.2 A W^−1^ and detectivity of 3.8 × 10^9^ Jones at 1 V. The MOF photodetector has excellent performance in both power and self-powered modes. In particular, this self-powered device demonstrates an ultrafast response time of 70 μs, which is the highest value of MOF-based photodetectors and is also competitive with inorganic and organic photodetectors. Furthermore, after bending at 180° for 1000 times, the performance of the flexible device remains constant.

Schottky junction is formed at the interface of metal and semiconductor, which is very useful in photodetectors due to its built-in electric field [111–113]. However, the low efficiency of photogenerated electron/hole separation and transport limits the application of Schottky junctions in self-powered devices. Xu et al. [79] overcome this issue by employing electronically conductive MOF (EC-MOF) materials into the junction. High-quality EC MOF thin films with flexible tunable crystal and electronic structure can be used to tune the Schottky junction of φ_B_. By inserting high-quality Cu_3_(C_18_H_6_(NH)_6_)_2_ thin film, the self-powered metal/n-Si Schottky diode was realized for the first time. Figure 8g shows the device structure and photoelectric performance of the Schottky diode and Fig. 8h shows the typical rectification characteristics, indicating the existence of Schottky junction in the device. The self-powered photodetector based on EC-MOF has high external quantum efficiency (84%) and a wide detectable spectral range (250–1500 nm), as well as short rise (0.007 s) and fall time (0.03 s).

Graphene, a single layer 2D honeycomb lattice has recently attracted great attention for use in the fabrication of flexible and stretchable devices because its outstanding optoelectronic property and distinct mechanical properties [84, 114]. Nevertheless, due to its weak light absorption (~ 2.3%), single-layer graphene cannot produce ultrahigh light response [115]. Recently, Chen et al. [84] combined the superior property of the MOF along with ultrahigh carrier mobility of graphene and firstly reported a highly sensitive, broadband, and wearable photodetector on a polydimethylsiloxane substrate. The external quantum efficiency of the hybrid photodetector is greater than 5 × 10^8^%. The porosity of MOF and graphene can help the light collection layer to capture photons, so the optical response of the device is > 10^6^ A W^−1^, and the response time is < 150 ms, which is about ten times faster than the current standard of graphene-organic hybrid photodetectors. In addition, due to the excellent flexibility of graphene layer, the device also has excellent flexibility [84]. Furthermore, in 2022, Chen et al. combined excellent features of a versatile MOF with monolayer graphene and report a self-powered ultrasensitive (external quantum efficiency ≈ 3 × 10^10^%) and ultrafast (response time ≈ 220 μs) wearable vertical phototransistor by utilizing a graphene/MOF/graphene/poly heterojunction on a flexible polydimethylsiloxane substrate [83].

**Fig. 9:**
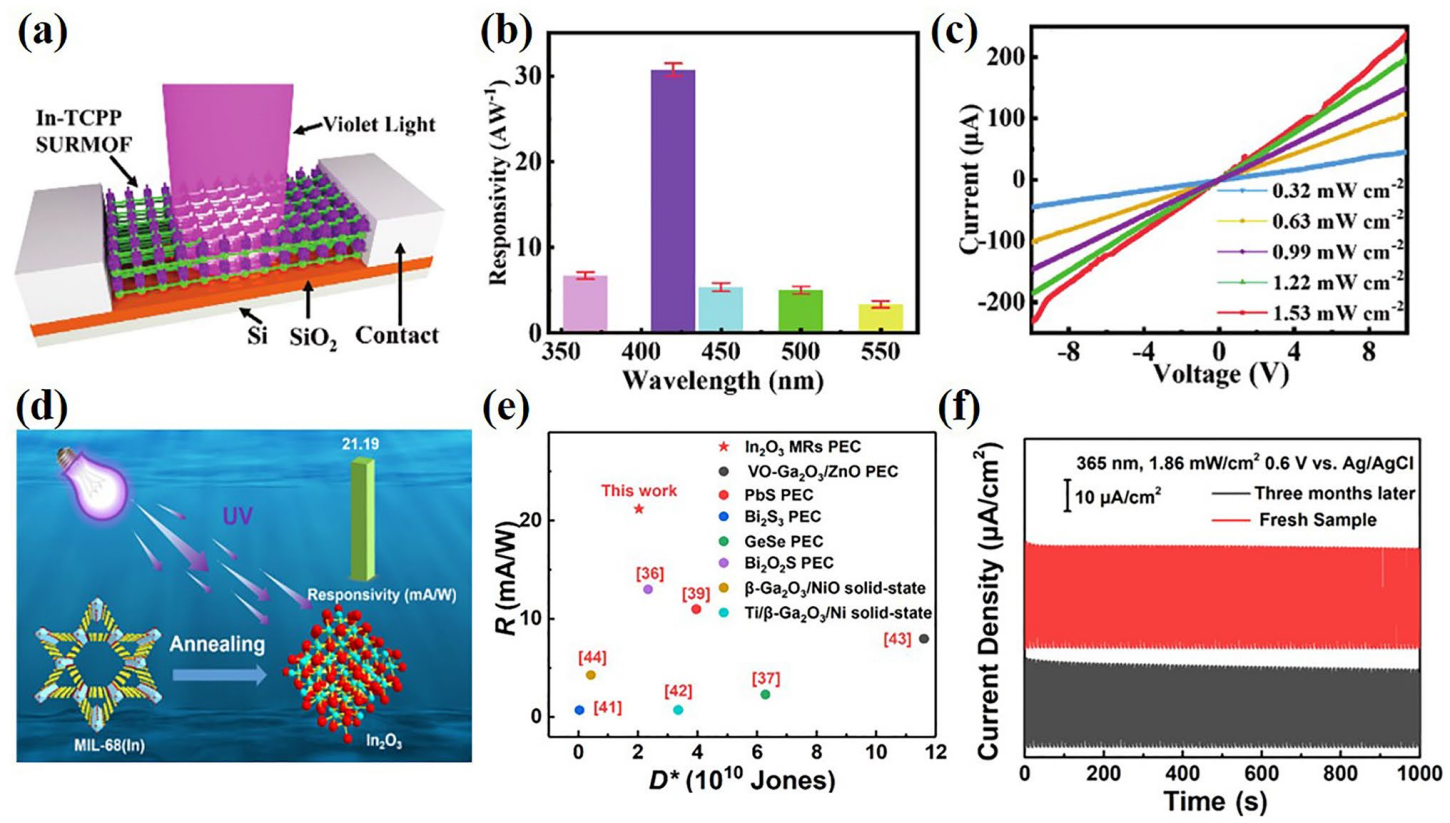
**a** Schematic diagram of In-TCPP SURMOF-based photodetector. **b** Responsivities versus light absorption of In-TCPP SURMOF at different wavelengths pulses irradiation; **c** Light intensity dependent I–V curves of MOF. Copyright 2021, WILEY–VCH [69]. **d** Schematic diagram of PEC UV PDs. **e** Comparison of PEC UV PD and several solid-state UV PD. **f** Multicycle and long-term stability of In_2_O_3_ MR PEC PDs irradiated by 365 nm for 100 cycles. Copyright 2022, American Chemical Society [119]

Porphyrins have the advantages of ultrafast electron injection, slow charge recombination kinetics, high absorption coefficient and good chemical stability under light [116, 117]. Gu et al. [69] reported a proof-of-concept photodetector assembled from an In-oxo chain-based metal-porphyrin framework thin film (Fig. 9a). By using liquid phase epitaxy, Monolithic, highly homogeneous In-TCPP SURMOF were grown on a functionalized substrate. The responsivity (*R*_λ_) is measured in the wavelength range of 365–600 nm ( Fig. 9b), and the recorded detection rate (D*) is 7.28 × 10^14^ Jones, short rise/fall time (0.07/0.04 s). And with the increase of irradiation intensity, the response current of the detector increases (Fig. 9c). This research provides a kind of oriented MOF thin film material for manufacturing high sensitivity photodetectors in the visible light region [69]. In addition, Gu et al. [118] reported the photodetector assembled with 2D metalloporphyrin-based MOF thin film. Due to the abundant π-π stacking between the MOF layers, the photodetector shows excellent photoresponse. In addition, the metalloporphyrin group in Zn-TCPP has an important influence on the photoresponse of the photodetector.

Some scientists also carry out research on photoelectrochemical photodetectors [119, 120]. Recently, Wang et al. [120] successfully synthesized a novel MOF host–guest material [Cd_3_(EtOIPA)_4_(HAD)_2_]·H_2_O under hydrothermal conditions. The MOF possesses a trinucleate Cd (II) based 2D double-layer with the protonated acridine (AD) cations as the template encapsulated into the grids. Photochemical measurements show that the high photocurrent density ratio under light and dark conditions is 290 at 0 V bias potential, making it a perfect self-driving photodetector. In 2022, Feng et al. [119] reported for the first time the photodetector of In_2_O_3_-based PEC ultraviolet PDs. Because of the charge transfer of semiconductors in PEC PDs, semiconductors with large specific surface area are conducive to manufacturing high-performance photodetectors (Fig. 9d). This device has good UV light response, showing a high response rate of 21.19 mA W^−1^ and 2.03 × 10^10^ Jones high specific detection rate (Fig. 9e). In addition, In_2_O_3_ MR PEC PD has good multi-circulation and long-term stability under 365 nm irradiation (Fig. 9f). Table 2 summarizes the device performance of UV–Vis photodetectors based on MOFs.

**Table 2:** Device performance of UV/Vis photodetector based on MOF

MOF	Metal; linker	Type	Preparation methodology	Wavelength (nm)	Detectivity (Jones)	Reference
Eu-MOF	Eu(NO_3_)_3_/H_2_BTC	Film	Spin-coating	254	1.015 × 10^10^	[109]
Zn-MOF	Zn(NO_3_)_2_/H_2_BDC	Crystal	Solvothermal	UVB	–	[42]
Cu_3_(HHTP)_2_	Cu(OAc)_2_/HHTP	Composites	Layer-by-layer of MOF on ZnO	365	3.8 × 19^9^	[110]
EC-MOF	Cu(OAc)_2_/HITP	Composites	Layer-by-layer on n-Si	450	–	[78]
Sr-MOF	Sr(NO_3_)_2_/1,4,5,8-naphth-alenetertacarboxylic	Composites	spin coated on top of graphene	325	6.9 × 10^14^	[83]
Sr-MOF	Sr(NO_3_)_2_/1,4,5,8-naphth-alenetertacarboxylic	Composites	spin coated on top of GrB/PVDF	530	4.7 × 10^8^	[82]
In-MOF	In(NO_3_)_3_/TCPP	Film	Layer-by-layer	420	7.28 × 10^14^	[117]
Zn-MOF	Zn(OAc)_2_/TCPP	Film	Layer-by-layer	420	8.1 × 10^13^	[118]
Cd-MOF	Cd_3_(EtOIPA)_4_(HAD)_2_]/ 5-ethoxyisophthalic acid	Crystal	Solvothermal	359	–	[119]
MIL-68	In(NO3)_3_/H_2_BDC	Crystal	Direct mixing of MOF with PVDF	365	2.03 × 10^10^	[120]

### NIR Photodetector

Infrared light is one of the many invisible rays in the sun's rays, which can be divided into three parts: near infrared (NIR), middle infrared (MIR), and far infrared (FIR). Among them, NIR with a wavelength range of 0.7–2.5 μm is crucial in a wide range of applications including optical communication, medical diagnosis, and military defense due to its unique and excellent performance [121–123]. However, the NIR photodetector based on MOF is still a challenge. Two-dimensional layered materials (2DLMs) are considered as the promising candidates for the next generation of high-performance infrared optoelectronic devices due to their unique structure and photoelectric properties [124, 125]. However, due to their poor optical absorption, the performance of 2DLM-based photodetectors cannot meet the requirements of practical applications [123].

**Fig. 10:**
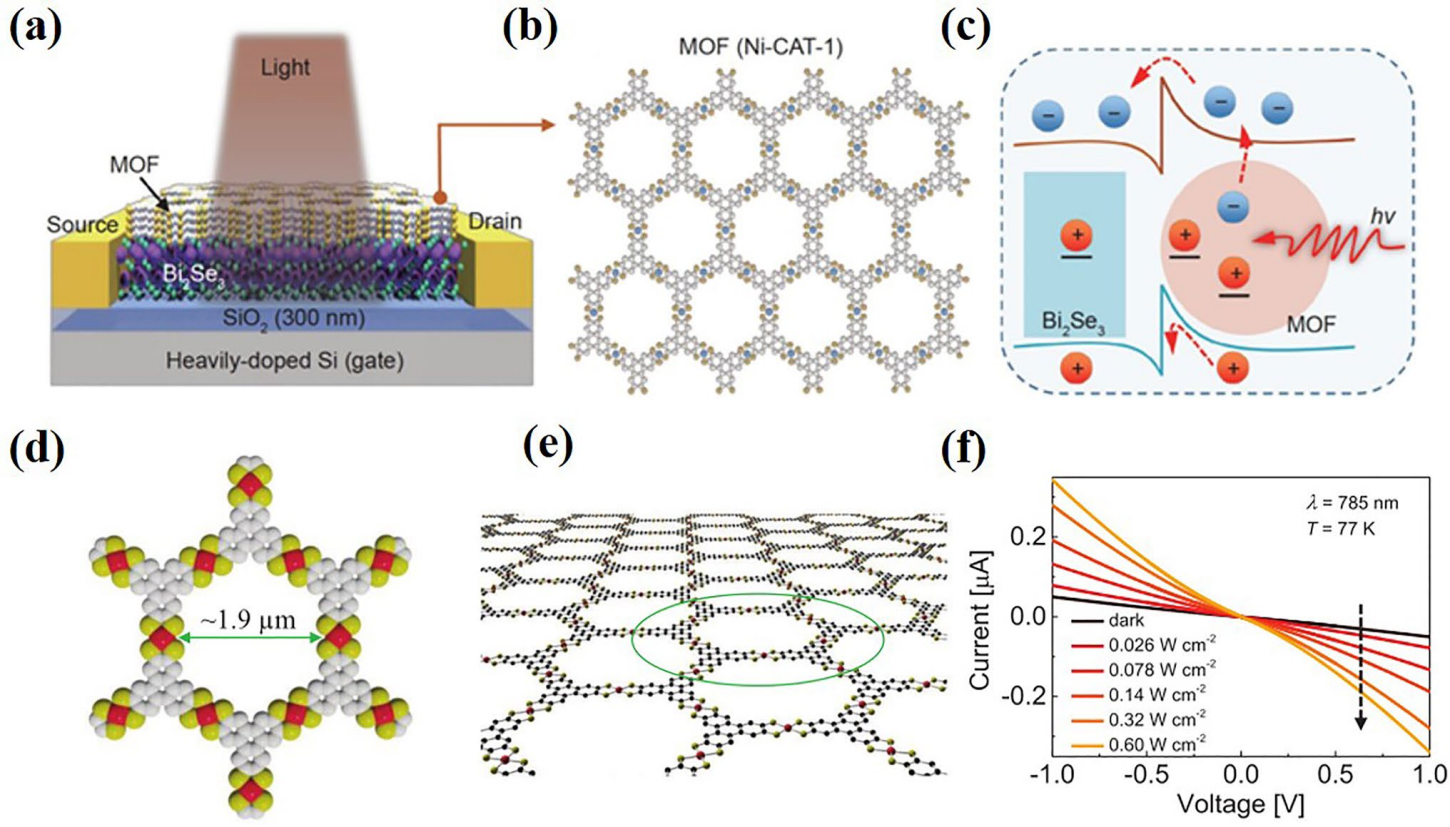
**a** Schematic diagram of the 2D Bi_2_Se_3_/MOF hybrid heterojunction; **b** Crystal structure of MOF (Ni-CAT-1); **c** Schematic band diagram of the heterojunction photodetector under illumination. Copyright 2021**,** Springer nature Limited [126]. **d** Chemical structure of the MOF film, Color code: red spheres represent iron atoms, yellow refers to sulfur atoms, and gray represents benzene rings. **e** Schematic of a monolayer of Fe_3_(THT)_2_(NH_4_)_3_ 2D MOF film; **f** I–V curves for different power densities of 785 nm wavelength at 77 K. Copyright 2020**,** WILEY–VCH [127]

Recently, Zhai et al. [126] proposed a strategy of combining MOF (Ni-CAT-1, Fig. 10b) nanoparticles with good optical absorption characteristics and 2DLM with high mobility to design high-performance near-infrared detectors (Fig. 10a). The photodetector presented a high responsivity of 4725 A W^−1^ and a superior detectivity of 3.5 × 10^13^ Jones at 1500 nm. The hybrid heterojunction has excellent performance due to the synergistic function of the enhanced optical absorption and photogating effect (Fig. 10c). In 2020, Erbe et al. [127] reported a photodetector device based on semiconducting Fe_3_(THT)_2_(NH_4_)_3_ two-dimension (2D) MOF thin films (Fig. 10d, e) operating in a broad spectral range. This device can detect a wide wavelength range from ultraviolet to near-infrared (400–1575 nm). Figure 10f shows the increased photocurrent with the increase of photon density, which proves that the active MOF layer acts as a photoconductor at this temperature. At 77 K, the device performance is significantly improved. Under 785 nm excitation, 7 × 10^8^ cm Hz^1/2^ W^−1^ has higher voltage response rate, lower noise equivalent power and higher specific detection rate. Owing to synthetic flexibility, large-area coverage, and cost-effective production of 2D conjugated MOF, these materials are promising candidates for a plethora of optoelectronic applications [127].

Quantum dots have been limited in the development of various photoelectric applications due to their poor chemical stability and weak electronic conductivity [128]. Lin and Fang et al. [129] synthesized a hybrid composite material (PbS@MOF) composed of PbS quantum dots and conductive MOF (Ni-HHTP) for short-wave infrared detection. The performance of PbS@MOF was evaluated by integrating PbS@MOF film into graphene field-effect transistor (FET) devices, achieving a high response rate of 301 A W^−1^ at ≈1400 nm. In addition, the presence of MOF significantly enhances the chemical stability of the quantum dots. Amorphous MOFs (aMOFs) possess advantages such as no crystal boundaries, isotropy, flexibility, and numerous defect-induced active sites compared to crystalline counterparts [130, 131]. However, aMOFs are typically synthesized under stringent conditions, and their properties and applications require further exploration. Gong et al. [132] synthesized a highly transparent p-type amorphous Cu-HHTP film (p-a-HHTP). By assembling it into a p-a-Cu-HHTP/n-Si self-powered heterojunction photodetector, they achieved an ultrafast photoresponse time of 40 μs and a near-infrared detection rate of 1.2 × 10^12^ Jones. In addition, p-a-Cu-HHTP and PD exhibit excellent high-temperature resistance. After heating the device and then cooling it to room temperature, there is no loss in performance.

Milichko et al. [133] achieved a single crystal photodetector with a spectral range of 600–1000 nm using HKUST-1 single crystals. HKUST-1 crystals undergo changes in their optical and electronic properties due to the desorption of water molecules when heated or photoinduced heating. The rapid and reversible desorption process of coordinated water molecules enables this photodetector to have a response rate of 0.1 s.

### Photoelectrochemical Detection in Bioanalysis

Photoelectrochemical (PEC) bioanalysis [134–136], a rapidly developing technique featured with reduced background and desirable sensitivity [137–142]. The PEC sensor technology combines optical radiation and electrochemical detection and has the advantages of both optical analysis and electrochemical sensing. Due to the completely separated forms of the excitation source (light) and detection signal (electrical signal), this method possesses reduced background interference and high detection sensitivity, which creates a substantial opportunity for advanced bioanalysis [44, 49, 143]. In 2012, Hu et al. [144] synthesized continuous film of MOF-5 on glassy carbon electrode (GCE). When ascorbic acid (AA) concentration changes from 0.4 to 1.5 mM, the signal response increases linearly. Porphyrin molecules constructed from porphyrin or metalloporphyrin ligands have prominent functions such as enzyme, biochemistry and photochemistry, Zhang et al. [145] developed a simple and fast PEC sensor based on zirconium porphyrin MOF (PCN-222) for the label-free detection of a phosphoprotein (α-casein). The sensor showed an enhanced photocurrent response to dopamine in an aqueous medium saturated with O_2_. Furthermore, Zhang et al. [146] developed a novel enzyme-free PEC immunoassay method based on nanoscale zirconium porphyrin MOF for the ultra-sensitive detection of prostate specific antigen (PSA). Under optimal conditions, the immunosensor possessed a wide calibration range of 1 pg mL ^−1^ to 10 ng mL ^−1^.

**Fig. 11:**
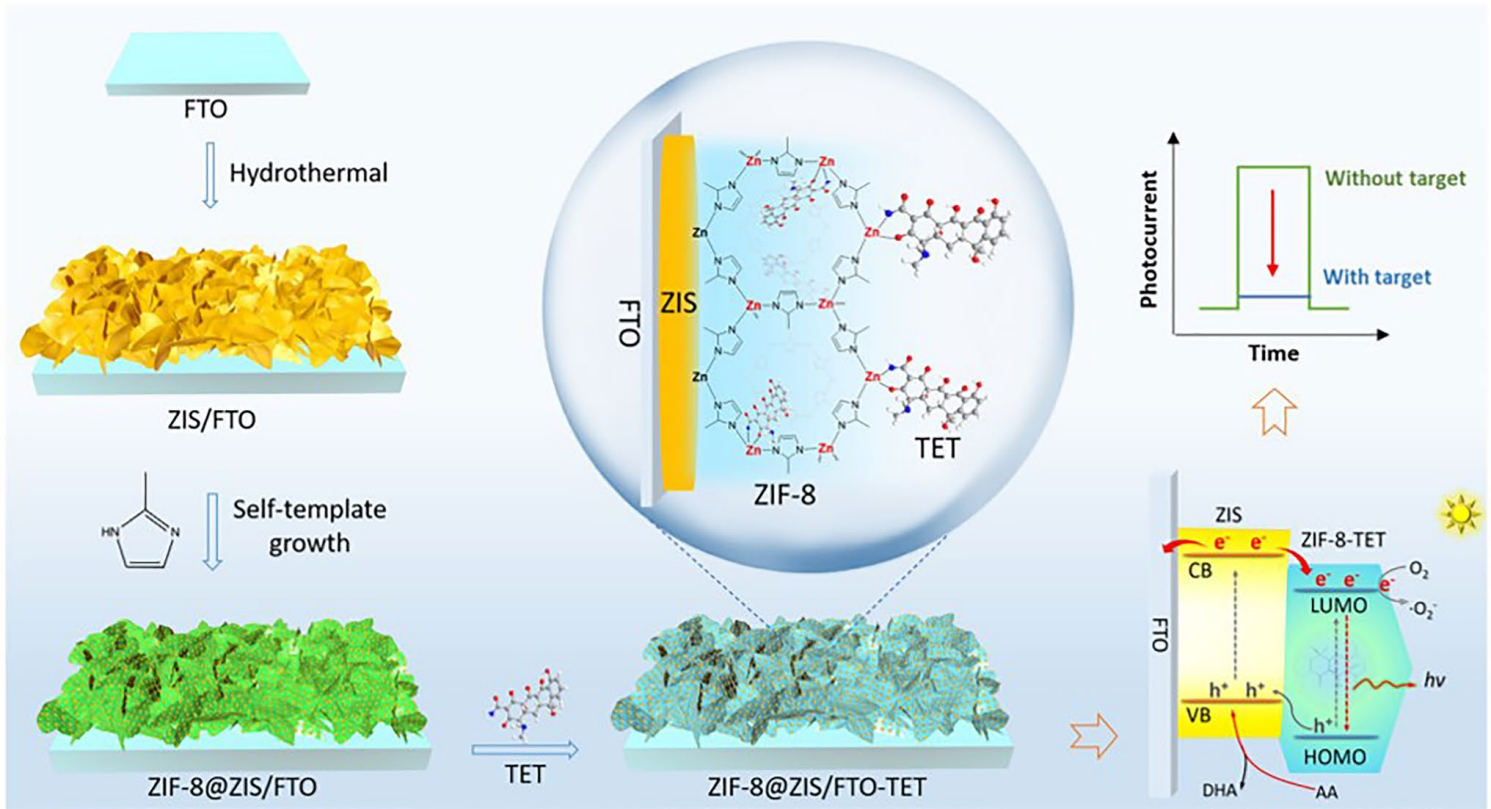
Fabrication of ZIF-8@ZIS/FTO for PEC Detection of TET. Copyright 2022 Elsevier Ltd [147]

Converting MOF into porous semiconductors is a newly emerged and efficient route to improve the conductivity and energy band structure of MOF [148, 149]. In 2021, Liu et al. [143] reported plasmon-promoted MOF-based PEC immunoassays with hierarchical structures. Due to the enhanced EM field and altering penetration depth of the EM field enabled by MOF shells, the AgAu/ZIF-8@ZnS@CdS exhibited ultrahigh photoactivity for PSA. The self-powered PEC sensing platform detecting PSA with LODs of 0.11 pg mL^−1^ and 0.9 fg mL^−1^. By using the same MOF (ZIF-8), Zhao et al. [147] reported a high-performance MOF-based PEC sensor toward tetracycline (TET). The ZIS/FTO was easily synthesized via a hydrothermal process, followed by in situ growth of ZIF-8 nanoshell (Fig. 11). Due to the interaction between TET and ZIF-8, the metal–organic complex structures would substantially quench the photocurrent signal by photoelectron transfer. In practical TET detection, the as-prepared sensor exhibited good performance in terms of exceptional speediness, ultralow detection limit, high stability, and high selectivity.

**Fig. 12:**
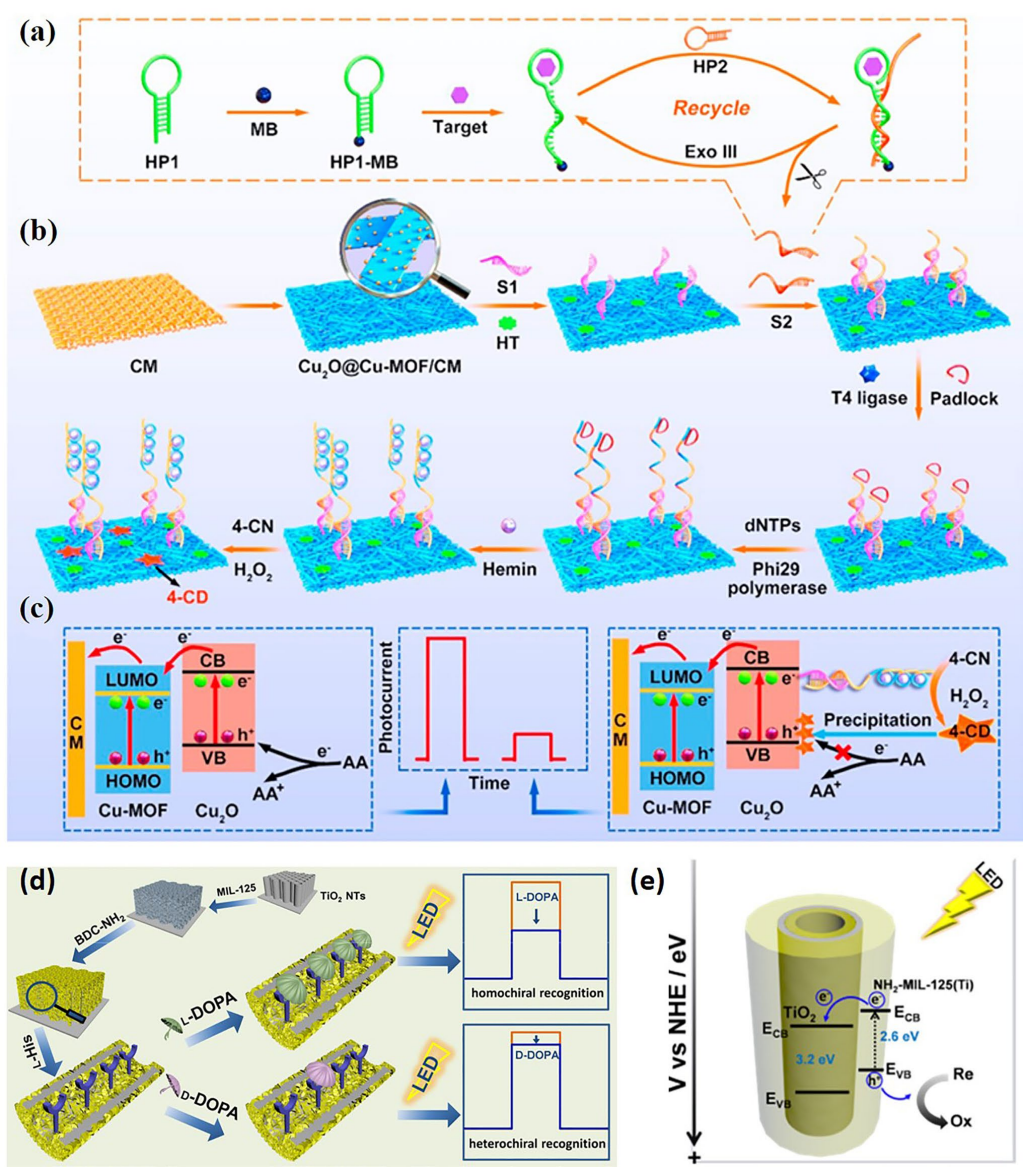
**a** Schematic diagrams of Exo III-assisted target cycle amplification strategy. **b** Preparation process of RCA based PEC aptasensor. **c** Mechanism on the charge-carrier transfer. Copyright 2020 Elsevier Ltd [151]. **d** Diagram of TiO_2_/MMIL-125-NH_2_ electrode manufacturing process. **e** The electron transfer process on the electrode under illumination. Copyright 2021, American Chemical Society [152]

The coupling of MOF and other functional materials has been proved to improve the properties of materials through synergy [135, 150]. Some metal oxides (ZnO, Cu_2_O, TiO_2_) exhibit excellent semiconductor properties in PEC applications [150]. In 2013, Kuang et al. [135] fabricated ZnO@ZIF8 nanorods and nanotubes with core− shell heterostructures. The as-prepared ZnO@ZIF-8 nanorod arrays display distinct photoelectrochemical response to hole scavengers with different molecule sizes (e.g., H_2_O_2_ and ascorbic acid) owing to the limitation of the aperture of the ZIF-8 shell. And the ZnO@ZIF-8 nanorod arrays have been successfully applied to detect H_2_O_2_ in slurry buffer solution. In addition, in 2020, Kuang et al. [151] reported a novel 3D nanobelt array structure Cu_2_O@Cu-MOF/CM prepared by in situ growth method (Fig. 12b, c). The detection sensitivity is greatly improved due to good cooperative signal amplification (Fig. 12a). This PEC aptamer sensor can be used for vascular endothelial growth factor 165 (VEGF_165_). The PEC sensor exhibited a wide calibration ranged from 10 to 1 × 10^8^ fM with a detection limit down to 2.3 fM (S/N = 3). And in 2021, Song et al. [152] integrated chiral amino acids and MOF into TiO_2_ NTs to develop enantioselective PEC platform for chiral recognition (Fig. 12d). When the visible light is irradiated, MIL-125-NH_2_ is excited, and then the electrons generated in the CB of MIL-125-NH_2_ are injected into the CB of TiO_2_, as shown in Fig. 12e. Because the greatly increased recognition sites by the homochiral MOF nanocrystals and the high PEC efficiency at the MIL-125-NH_2_/TiO_2_ interface, the obtained chiral MOF-in-NT electrode showed excellent sensitivity and enantioselectivity for the recognition of 3,4-dihydroxyphenylalanine (DOPA) enantiomers.

**Fig. 13:**
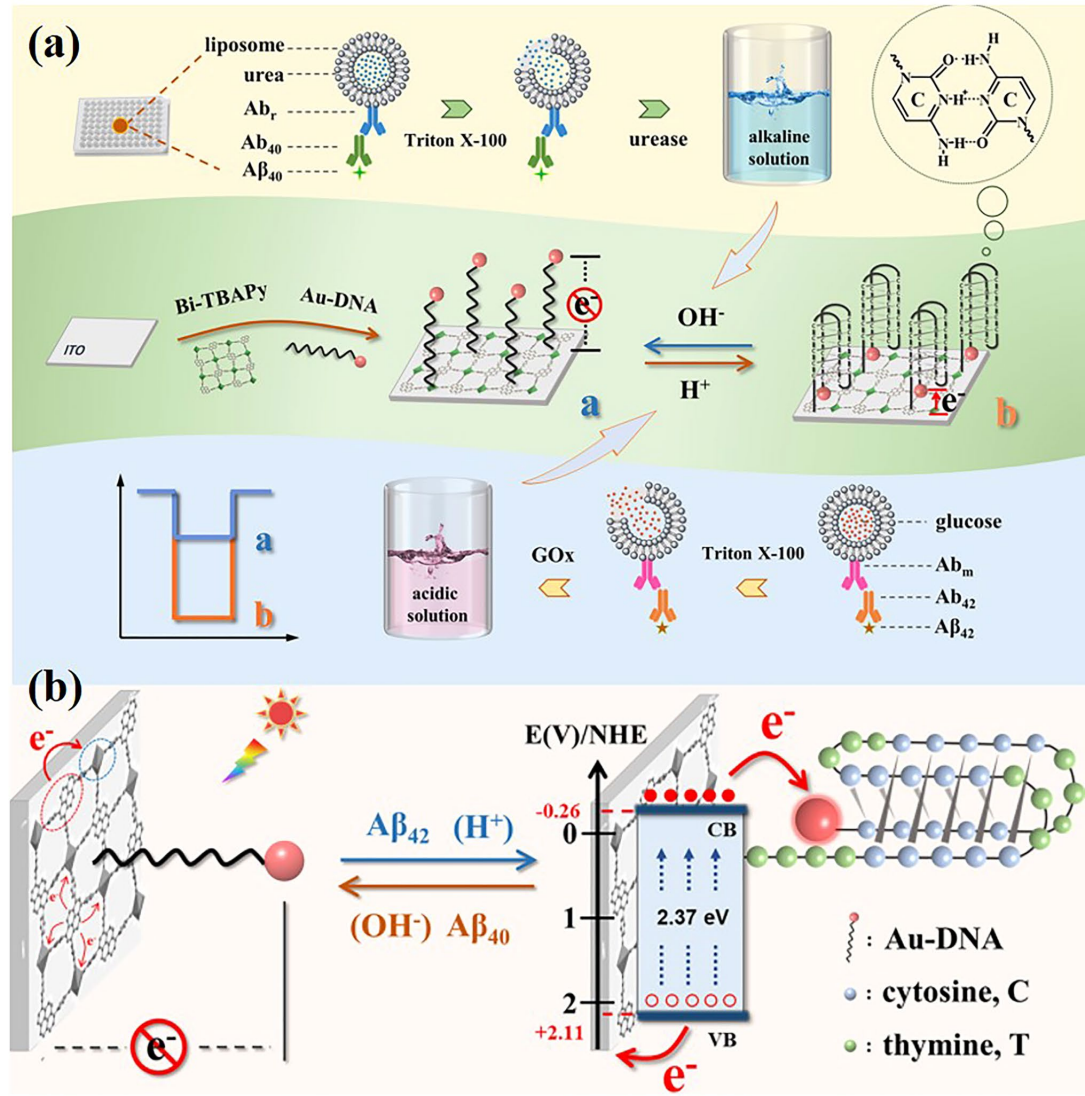
**a** Schematic of switchable multi-channel PEC sensing mechanism. **b** Electron transfer mechanism of the switchable PEC sensor under light. Copyright 2022, American Chemical Society [153]

The electron-transfer tunnelling distance between noble metals and photoactive materials has an important influence on electron transfer tunnelling effect. Thus, it is feasible to adjust the PEC signal by adjusting the tunnel distance. On this basis, Song et al. [153] report an I-motif-based switchable sensing approach to construct a PEC immunosensor for Aβ_42_ and Aβ_40_ by using Bi-TBAPy as an efficient photoactive cathode material (Fig. 13a). Figure 13b describes the sensing principle of the switchable multi-channel PEC sensor. In the presence of Aβ_42_, split-type biorecognition produces the acidic solution, which triggers the formation of the i-motif structure. PEC analysis of switching multiple targets on one interface is realized by adjusting the electron transfer tunnel distance between Au NPs and bi-copy. In addition, the PEC sensor has the advantages of simple operation, high accuracy and strong versatility.

In the construction of PEC biosensors, the reasonable design of photoactive materials and the rational planning of signal amplification strategies are particularly important. Exploiting the photoactivity and strong binding properties of PCN-224, Lin et al. [154] used solvothermal method to grow PCN-224 on graphene to obtain a PCN-224/RGO (reduced graphene oxide) composite. RGO can improve the photogenerated electron hole separation and realize synergistic amplification with PCN-224 photocurrent signal. In 2019, Li et al. [155] prepared ZnS/C/MoS_2_ nanocomposites based on MOF for photoelectric sensing. ZnS/C/MoS_2_ nanocomposites have enhanced photocurrent response. Under light, ascorbic acid (AA) can react with the photogenerated holes of ZnS/C/MoS_2_ nanocomposites to generate photocurrent for quantitative analysis. The PEC immunosensor has excellent performance with a linear range of 2.0 pg mL^−1^ to 10.0 ng mL^−1^ and a detection limit of 1.30 pg mL^−1^ (S/N = 3).

### Circularly Polarized Light Detection

Polarization is an inherent property of light, and circular polarized light (CPL) detection is of great significance in quantum computing, optical communication, quantum optics and other fields [156, 157]. Traditional circularly polarized light detection methods involve multiple optical components, making it challenging to implement micro-integrated CPL detectors. To achieve this, the material must exhibit handedness photoelectric properties to generate different excitations when absorbing left-handed CPL (LCP) and right-handed CPL (RCP) photons [158]. Chiral materials exhibit different absorption of LCP and RCP, so photodetectors based on chiral materials can directly detect CPL. In 2023, Chen et al. [159] developed chiral metal–organic framework (CMOF) based on non-chiral building blocks as efficient spin polarization flexible photodetectors. In the application of spin polarization flexible detector, the detection rate (D*) is 1.83 × 10^12^ Jones, and the anisotropy factor (g_lph_) detected by CPL is 0.38. CMOF has high optical responsiveness and optical gain.

**Fig. 14:**
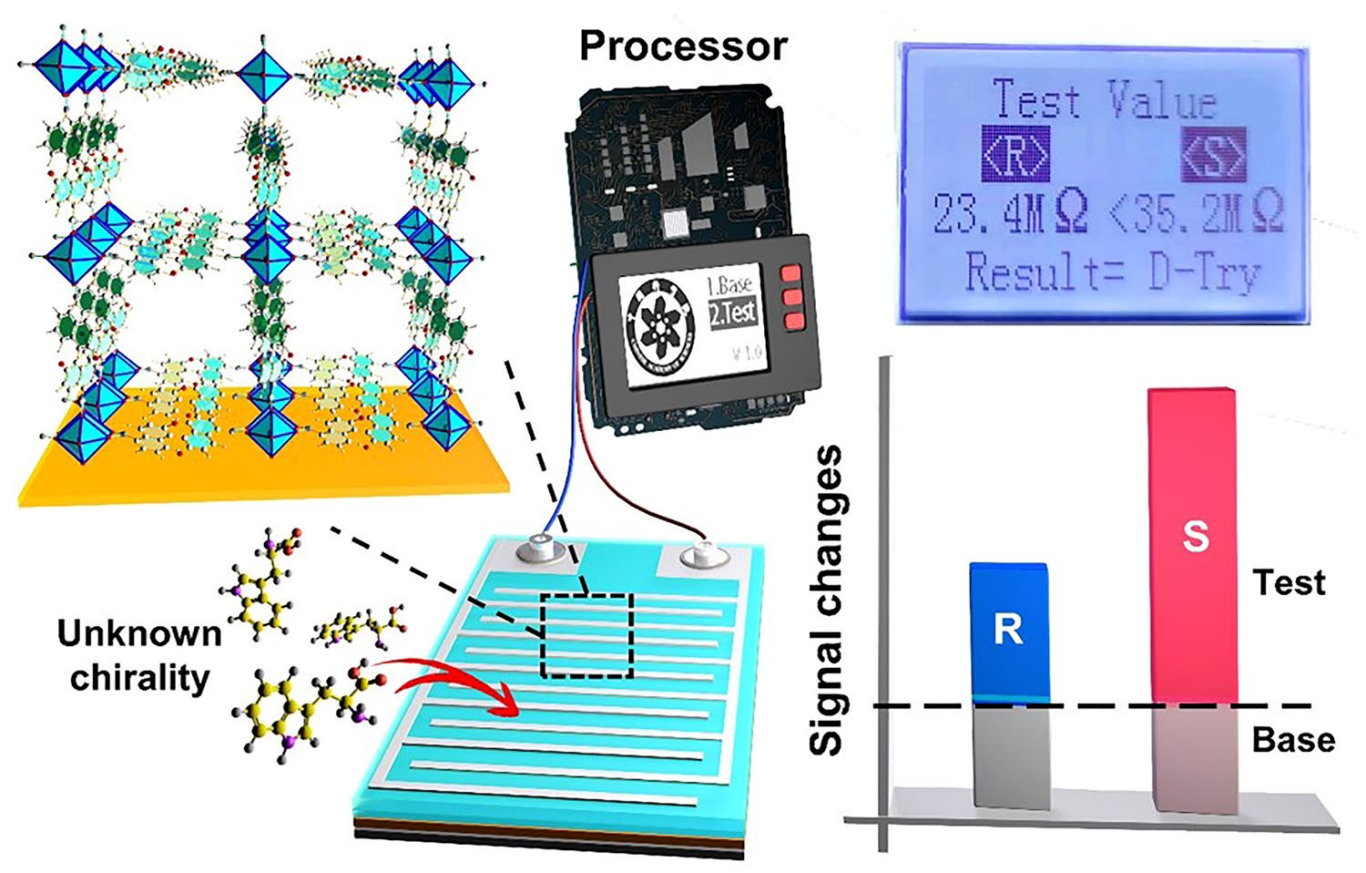
Schematic diagram of the device for detecting enantiomers through signal analysis. Copyright 2023, American Chemical Society [161]

Based on the structural tunability of MOF, chiral ligands can be directly selected to give MOF chirality for circularly polarized light detection. Lars et al. [160] used the homochiral BINOL group to functionalize porphyrin molecules. The porphyrin part is planar when dissolved. However, by assembling with zinc acetate to form SURMOF, the porphyrin part acquired chirality. A chirality dependent photoconduction of circularly polarized light with an asymmetry coefficient g of 4.3 × 10^–4^ is obtained. This assembly-induced chirality will enable a large number of organic materials to have excellent chiral and optoelectronic properties. Gu et al. [161] obtained a pair of chiral SURMOF with orientation using layer by layer method. Further integration of SURMOF into highly sensitive photodetectors resulted in an anisotropic factor of up to 0.41. In addition, SURMOF exhibited significant differences in the uptake of amino acid enantiomers (Fig. 14). By using CP light to manufacture D-and L-neneneba tryptophan sensor devices, it shows that MOF film has great potential in chiral analysis. This work opens up a new direction for the future application of devices based on chiral MOF materials.

## Conclusions and Outlook

In this review, the most recent research progress of photodetectors based on MOF materials are discussed. We summarized the preparation, photodetectors performance and applications of MOF-based photodetectors reported in the latest literatures. Firstly, the methods of assembling MOF-based photodetectors and various types of MOF-based materials (including single crystals, thin films, and MOF composites) are detailed in this review. Additionally, the applications of these materials in X-ray detectors, ultraviolet detectors, infrared detectors, biological detectors, and circularly polarized light photodetectors are discussed. These discussions illustrate the great potential for the integration of semiconductor MOF in active devices. In terms of logical design and optimization of its periodic structure, physical and chemical characteristics, MOF is structurally more adaptable than conventional inorganic or organic materials. Semiconductor MOF is becoming an increasingly promising material for photoelectric detection. Although considerable achievements have been made, MOF-based photodetectors are still in their infancy, and have a certain gap in some aspects compared with traditional photodetectors. Therefore, in order to realize the commercial application of high-performance semiconductor MOF and corresponding electronic devices as soon as possible, challenges need to be overcome from the following aspects:

Assembling MOF into uniform dense films. It is well known that although single crystals have high mobility, they also have the disadvantages of rigidity and brittleness, which greatly limits their further application in devices. This problem can be solved perfectly by assembling MOF into thin films. In addition, different functional applications can be achieved by growing the film on different substrates. However, only a portion of MOF can grow into uniformly dense films. There are also issues related to device integration, compatibility between MOF-related processes and substrate materials.

MOF is generally considered to be insulating materials with a high band gap. To achieve high efficiency semiconductor MOF, the advantages of tunable structure of MOF can be utilized by a) increasing conjugation of bridging ligands; b) selecting electron-rich metal nodes and organic ligands; c) to functionalize ligands with nitro or amino groups to reduce the band gap. The light absorption in MOF is determined by the energy difference between organic ligands and metal ions. Adjusting different metal ions/organic ligands can effectively regulate the light absorption of MOF. Of course, introducing different materials into the MOF framework also changes electronic properties and promotes light absorption. Furthermore, regulating the semiconductor energy gap or developing heterostructures can effectively regulate the semiconductor absorption wavelength range. At present, only a few photoactive MOF materials can be used for photodetectors, because the available ligands are mainly porphyrins, anthracene nuclear derivatives, naphthalene nuclear derivatives and thiophene.

- Optimize the stability of the device under environmental conditions. Due to the inherent electrochemical sensitivity of MOF materials, it is urgent to optimize the operating stability of the device. The current methods used in the laboratory to synthesize MOF single crystals and thin films are difficult to apply to large-scale manufacturing, resulting in poor reproducibility between batches and a large amount of material waste, which is not conducive to the development of low-cost commercialization. Therefore, the production technology of large MOF films needs to be further improved.
- Explore and develop new multifunctional intelligent active devices based on semiconductor MOF. If more performance can be achieved with less material, the cost can be greatly reduced. Furthermore, the future endeavors in the realm of MOF materials research ought to encompass not merely the optimization of performance metrics but also the meticulous design and synthesis of environmentally benign or non-biohazardous MOFs. For instance, a pivotal direction should involve delving into the development of eco-friendly ligands for the construction of semiconductor MOFs, with the ultimate goal of fabricating non-toxic and cost-competitive photodetectors. This dual-pronged approach ensures not only advancements in technological capability but also aligns with sustainable development principles.

In summary, the application of high efficiency semiconductor MOF and their devices is summarized and discussed in this paper. We hope that this review will provide a comprehensive and critical reference for the field and contribute to the technology maturity and commercialization of high-performance MOF semiconductor materials.
